# The diversity and breadth of cancer cell fatty acid metabolism

**DOI:** 10.1186/s40170-020-00237-2

**Published:** 2021-01-07

**Authors:** Shilpa R. Nagarajan, Lisa M. Butler, Andrew J. Hoy

**Affiliations:** 1 Discipline of Physiology, School of Medical Sciences, Charles Perkins Centre, Faculty of Medicine and Health, The University of Sydney, Sydney, NSW Australia; 2 Oxford Centre for Diabetes, Endocrinology and Metabolism, Radcliffe Department of Medicine, University of Oxford, Churchill Hospital, Oxford, UK; 3 Adelaide Medical School and Freemasons Foundation Centre for Men’s Health, University of Adelaide, Adelaide, SA Australia; 4 South Australian Health and Medical Research Institute, Adelaide, SA Australia

**Keywords:** Fatty acid, Lipid, Cellular membrane, De novo synthesis, Lipid droplets, Mitochondria, Peroxisome, Oxidation

## Abstract

Tumor cellular metabolism exhibits distinguishing features that collectively enhance biomass synthesis while maintaining redox balance and cellular homeostasis. These attributes reflect the complex interactions between cell-intrinsic factors such as genomic-transcriptomic regulation and cell-extrinsic influences, including growth factor and nutrient availability. Alongside glucose and amino acid metabolism, fatty acid metabolism supports tumorigenesis and disease progression through a range of processes including membrane biosynthesis, energy storage and production, and generation of signaling intermediates. Here, we highlight the complexity of cellular fatty acid metabolism in cancer, the various inputs and outputs of the intracellular free fatty acid pool, and the numerous ways that these pathways influence disease behavior.

## Background

Cancer cells have distinctive metabolic phenotypes compared to normal, non-malignant cells that are characterized by altered nutrient metabolism that supports the rapid manufacture of biomass, all while managing redox balance (see reviews [1, 2]. Our understanding of cancer metabolism is becoming more sophisticated with the identification of metabolic heterogeneity within cancer types [3–9], and that some aspects of cellular metabolism differ between low-grade and high-grade disease [10–13], as well as between primary tumors and metastatic tissue [14, 15]. In general, these observations arise from the assessment of cancer cell glucose (i.e., the Warburg Effect; see reviews [2, 16]) and amino acid metabolism including glutamine (see reviews [2, 17]), proline [18], and methionine [19, 20]. Broadly speaking, alterations in lipid metabolism associated with tumorigenesis and promotion of tumor progression is comparatively less well defined than these other nutrients and their metabolic pathways.

Lipids are a broad church of hydrophobic biomolecules that participate in a wide array of metabolic pathways. A consensus view is that highly proliferative cells, such as cancer cells, require the lipid building blocks for membrane synthesis, as well as other biomass, to support replication [21]. However, lipids can influence cancer cell biology via a range of mechanisms, which include but are not limited to, fatty acids as substrates for mitochondrial ATP synthesis [22], arachidonic acid (20:4(ω-6)) as the pre-cursor for eicosanoid synthesis (see review [23]), post-translational protein-lipid modifications of signaling proteins [24], and cholesterol as a substrate for de novo steroidogenesis in prostate cancer [25]. Additionally, lipid composition influences the physicochemical properties of cellular membranes and can modulate protein function [26]. A key example is the well-defined influence that phosphoinositide 3-kinases (PI3K) have on cell biology by phosphorylating the membrane lipid phosphatidylinositol (4,5)-bisphosphate to phosphatidylinositol (3,4,5)-trisphosphate (PI(3,4,5)P3). In contrast, phosphatase and tensin homolog (PTEN) catalyzes the reverse reaction [27]. The presence of PI(3,4,5)P3 in the plasma membrane leads to the recruitment and binding of phosphoinositide-dependent kinase-1 (PDK-1), which results in the activation of Akt and mTORC2 and downstream biological events including mitogenic and metabolic endpoints [28]. Similarly, phosphatidate (an intermediate of the Kennedy/glycerolipid synthesis pathway (see below)) regulates mitogenic mTOR complex signaling [29–31] and LKB1 signaling [32]. These examples highlight the broad influence lipids have on cancer cell biology beyond the requirements for glycerophospholipids to produce new membranes for replication. In fact, due to their role in many aspects of cell biology, it has been proposed that lipids can regulate many of the Hallmarks of Cancer (see review [33]).

Recently, there have been significant advances in our understanding of fatty acid metabolism and its role in many aspects of cancer cell biology that influence disease behavior. Here, we take a holistic view of fatty acid metabolism by defining the diverse extracellular and intracellular inputs to the free fatty acid pool and the numerous outputs. Alongside this summary, we describe the role that tumor fatty acid metabolism plays in cancer progression reported during the past 5 years.

## Tumor fatty acid metabolism pathways and their role in cancer progression

Fatty acid species vary in the number of carbons and the number of double bonds that they contain, which impacts on the chemical and bio-physical properties of the fatty acids as well as those complex lipids that use fatty acids as building blocks. In general, long-chain fatty acids have carbon (C) chain lengths of 12–20, and very long-chain fatty acids have C ≥ 22. Fatty acids are also categorized into saturated fatty acids, monounsaturated fatty acids (MUFAs), and polyunsaturated fatty acids (PUFAs) as determined by the number of double bonds. This diversity of fatty acid length and saturation leads to the potential for more than 10 000 distinct lipid species of various lipid classes (i.e., glycerophospholipids, glycerolipids, sphingolipids, sterols, etc.) to exist in mammalian cells [34]. That said, this number will be lower as not all possible fatty acids occur and there is a positional preference for fatty acids in lipids; the sn-1 position tends to have a shorter more saturated fatty acid, whereas the sn-2 position has a longer and more unsaturated fatty acid. In any event, the diversity of fatty acid species introduces specificity in certain key metabolic pathways, especially catabolic pathways.

**Fig. 1:**
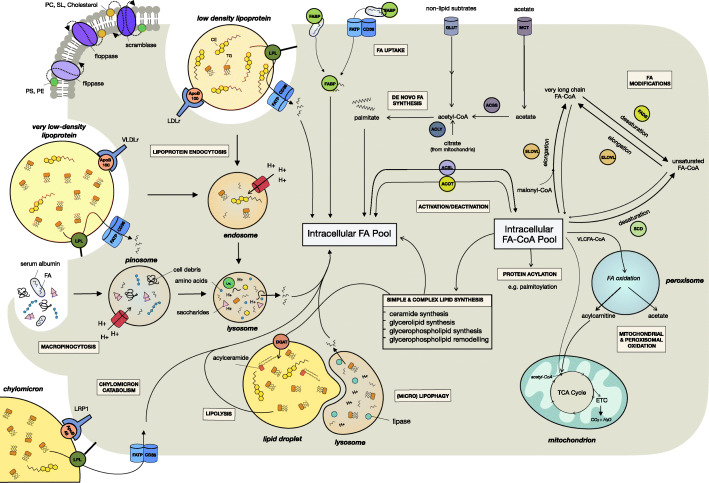
Fatty acid uptake, synthesis, and metabolism pathways. Overview of the extracellular sources of fatty acids, including chylomicrons, VLDL and LDL lipoproteins, albumin-bound free fatty acids, and macropinocytosis, and the intracellular pathways that contribute to the intracellular fatty acid pool. Intracellular sources of fatty acids include de novo fatty acid synthesis from non-lipid substrates, lipid droplet lipolysis and lipophagy, fatty acyl-CoA, and phospholipid hydrolysis. Fatty acids are converted to fatty acyl-CoAs that are substrates for a range of reactions including elongation and desaturation, glycerolipid, glycerophospholipid and sphingolipid synthesis, protein acylation, and oxidation in peroxisomes and mitochondria. *ACOT* acyl-CoA thioesterases, *ACLY* ATP citrate lyase, *ACSL* long-chain acyl-CoA synthase, *ACSS* cytoplasmic acetyl-CoA synthetase, *CD36* cluster of differentiation 36, *CE* cholesteryl ester, *DGAT* diacylglycerol acyltransferase, *ELOVL* elongation of very long-chain fatty acid enzymes, *ETC* electron transport chain, *FABP* fatty acid binding protein, *FA*-*CoA* fatty acyl-CoA, *FA* fatty acid, *FADS* fatty acid desaturases, *FATP* fatty acid transport protein, *GLUT* glucose transporter, *LDLr* low-density lipoprotein receptor, *LPL* lipoprotein lipase, *LPR1* low-density lipoprotein receptor-related protein 1, *MCT* monocarboxylate transporter, *PC* phosphatidylcholine, *PE* phosphatidylethanolamine, *PS* phosphatidylserine, *SCD* stearoyl-CoA desaturase, *SL* sphingolipid, *TG* triacylglycerols, *VLCFA*-*CoA* very-long chain fatty acyl-CoA, *VLDLr* very low-density lipoprotein receptor

In this review, we will put forward our views of the breadth and complexity of tumor fatty acid metabolism, focusing on long-chain fatty acid metabolism and discussing its pathways in generalities. By focusing on long-chain fatty acids, we will not discuss short-chain fatty acid metabolism (see review [35]) or the many fatty acid variants that exist, including epoxide-modified, branched-chain, and nitro-fatty acids (see reviews [36–38]). We have structured our review in an intracellular fatty acid-centric manner—defining the inputs and the outputs of this pool, and the role that these pathways play in cancer cell biology. By doing so, we have excluded related aspects of lipid metabolism, such as modifications of complex lipids that include phosphorylation and hydrolysis of head-groups, that can influence cancer cell biology. Tumor hypoxia affects many aspects of metabolism, including lipid metabolism [39, 40] and interested readers are pointed to a recent review on this specific topic (see review [41]). We also want to acknowledge that fatty acid metabolism is intimately entangled with carbohydrate and amino acid metabolic pathways, with many sharing intermediates (Fig. 1) and due to space constraints, we will only point to a very limited number of interactions. There is no doubt that in our attempts to be comprehensive in the breadth of our review that we have oversimplified many facets of lipid metabolism, and we highlight many excellent reviews that dive much deeper into these areas.

### Inputs of the intracellular fatty acid pool

The intracellular fatty acid pool is the source of building blocks for complex lipids and mitochondrial oxidative metabolism (Fig. 1; see the “Outputs of the intracellular fatty acyl-CoA pool and their influence on cancer cell behavior” section). This pool has many intracellular and extracellular supply sources; however, it should be noted that the stoichiometric relationships between these diverse sources remain to be defined.

#### Extracellular fatty acids

##### Protein-mediated uptake

The extracellular pool of fatty acids consists of several sources. They include those in the plasma: adipocyte-derived, albumin-bound free fatty acids (or non-esterified fatty acids), and those contained in lipoprotein triacylglycerols and/or fatty acid esters (i.e., cholesteryl esters) and glycerophospholipids. These lipoprotein-contained fatty acids can be liberated by the actions of extracellular lipases, including lipoprotein lipase (LPL) and secreted phospholipase A_2_ [42]. Likewise, there are stromal supplies which include local adipocytes, cancer-associated fibroblast-derived extracellular vesicles [43], and may consist of autophagy-lipophagy of stromal cells (analogous to the transfer of cancer-associated fibroblast-derived amino acids, etc. [44]). Finally, extracellular lysophospholipids can be taken up by cells; however, the mechanism by which these lipids cross the plasma membrane remains to be defined [45].

In general, extracellular sources of fatty acids are taken up by cells via two mechanisms:

A. Extracellular free fatty acids, including adipocyte-derived or liberated by extracellular lipases, are transported into cells via membrane-associated proteins including scavenger receptor B2 (SR-B2; also known as cluster of differentiation 36 (CD36)), fatty acid transport proteins (FATPs), and plasma membrane fatty acid-binding protein (FABP; see review [46]) or via passive diffusion [47]. There remains significant debate regarding the role that protein-mediated uptake versus passive diffusion plays in free fatty acids uptake by cells.
B. Fatty acids contained in triacylglycerol-rich chylomicrons and very-low-density lipoproteins (VLDL) or cholesteryl ester-rich low-density lipoproteins (LDL) can be endocytosed via the actions of receptors including VLDL-receptor (VLDLr), LDL-receptor (LDLr), lipolysis-stimulated receptor (LSR) [48], low-density lipoprotein receptor-related protein 1 (LPR1) [49], or SR-B1 [50]. These fatty acid-rich particles then enter the endosomal-lysosomal pathway involving lysosomal acid lipases to liberate free fatty acids [51].

To date, few studies have assessed the rate of long-chain free fatty uptake in cancer cells. One notable study reported that malignant prostate cancer tissue had higher fatty acid uptake rates compared to patient-matched benign tissue [52]. Interestingly, the same authors reported that near-complete ablation of SR-B2/CD36 mRNA reduced free fatty acid uptake by only 35% in PC-3 prostate cancer cells. This is consistent with knockdown of SR-B2/CD36 in SKOV3ip1 ovarian cancer cells which attenuated fatty acid uptake by ~ 40% [53]. In vivo, ablation of SR-B2/CD36 in the prostate tissue of Pten-deficient mice reduced fatty acid uptake by ~ 55%, while treating mice harboring PDXs of localized high-risk prostate cancer with a SR-B2/CD36 mAb reduced fatty acid uptake by 22% [52]. These studies demonstrate that SR-B2/CD36 plays a role in extracellular fatty acid uptake in cancer cells, but other mechanisms contribute to this process.

Despite this quantitatively “minor” role in fatty acid uptake, recent loss-of-function studies have clearly shown that SR-B2/CD36 is critical in prostate [52], ovarian [53], oral [54], cervical [55], gastric [56], breast [57, 58], and liver [59] cancer biology. For example, prostate-specific deletion of SR-B2/CD36 of cancer-susceptible Pten^−/−^ mice slowed cancer progression, while SR-B2/CD36 antibody therapy reduced cancer severity in patient-derived xenografts [52]. Likewise, SR-B2/CD36^+^ human oral carcinoma cells initiate metastasis in an SR-B2/CD36-dependent manner, and SR-B2/CD36 antibody therapy inhibited metastasis in preclinical models [54]. An array of signaling pathways that mediate these patterns have been proposed (see review [60]); however, the precise mechanism by which the reduced uptake of extracellular fatty acids by SR-B2/CD36 influences cancer cell biology remains a mystery.

Compared to SR-B2/CD36, the other proposed regulators of fatty acid uptake, such as FATPs and FABP plasma membrane [46], have received far less attention. This is likely due to what appears to be a widely held belief that SR-B2/CD36 is rate-limiting in fatty acid uptake or the only fatty acid transporter. Recently, Zhang and colleagues demonstrated that FATP1, which is overexpressed in melanoma, is required for fatty acid uptake and melanoma growth and invasion in vitro and in vivo [61]. What role the other five members of the FATP family play in cancer biology remains an area of opportunity.

The contribution of lipoprotein particles to the intracellular fatty acid pool remains poorly defined within cell biology. One key reason could be that triacylglycerol-rich chylomicrons and VLDL can provide fatty acids via at least two distinct pathways—extracellular lipolysis to liberate free fatty acids for uptake, or endocytosis [62]. Despite this, it is clear that cells, including U87-MG glioblastoma cells and MDA-MB-231 breast cancer cells, increase intracellular lipid levels and VLDL uptake in a time-dependent and dose-dependent manner [63, 64]. Triacylglycerol-rich VLDL can be endocytosed by VLDLr and protein levels of VLDLr are increased in hepatocellular carcinoma [65] and clear-cell renal cell carcinoma [66] compared to adjacent non-cancerous tissue. Knockdown of VLDLr reduced lipoprotein uptake and intracellular lipid levels in clear-cell RCC cells [66], and in MCF-7 and MDA-MB-231 breast cancer cells [64, 65]. To further complicate our understanding, it was recently demonstrated that VLDLr-mediated VLDL uptake requires LPL acting non-catalytically to facilitate endocytosis [64]. Triacylglycerol-rich lipoproteins are also metabolized by the cell surface protein LSR, which is highly expressed in breast cancer [67]. However, LSR’s role in breast cancer metabolism is complicated as it also regulates tight junctions and was recently identified to be capable of nuclear localization and DNA binding [67]. Likewise, LPR1, which is involved in lipoprotein transport, ligand uptake, and receptor-mediated endocytosis, also regulates cell surface protease activity and acts on many cell signaling pathways, and so its role in lipid-mediated changes in cancer biology is very complex [68]. Finally, the role of LDLr and its related proteins in cancer biology has rightly centered on its role in cholesterol homeostasis, and not fatty acid metabolism; however, it is conceivable that the glycerophospholipids and the fatty acid of cholesteryl esters contribute to intracellular fatty acid levels. To date, the contribution of this pathway to the intracellular fatty acid pool remains unknown.

##### Macropinocytosis

A likely alternative pathway for the accumulation of extracellular fatty acid-based lipids is macropinocytosis. While this is a known mechanism for cancer cells to acquire extracellular proteins which then are processed by the endosomal-lysosomal pathway (see review [69]), it is conceivable, and has been hypothesized [69, 70], that lipids, including fatty acid-based lipids, are also endocytosed and contribute to the intracellular fatty acid pool. In fact, supplying mouse mPCE or human DU145 prostate cancer cells necrotic cell debris in glucose- and amino acid-restricted media completely restored lipid droplet content, suggesting membranes and lipids present in necrotic debris can maintain lipid stores [71].

#### Intracellular fatty acids

##### De novo synthesis

The synthesis of new long-chain fatty acids from non-lipid substrates is another input to the intracellular free fatty acid pool. We will focus on the cytosolic pathway but acknowledge that mitochondria are capable of synthesizing predominantly short- and medium-chain fatty acids that can act as precursors for lipoic acid synthesis and protein lipoylation [72, 73]. The cytosolic synthesis of fatty acids starts with the export of mitochondrial citrate into the cytosol via the mitochondrial tricarboxylate transporter (encoded by SLC25A1) where it is converted into acetyl-CoA by ATP-citrate lyase (encoded by ACLY; Fig. 1). Extra-mitochondrial acetyl-CoA is also generated by acyl-coenzyme A synthetase short-chain family member 2 (ACSS2) [74] from acetate, which itself can be derived from a range of sources including extracellular acetate and the recently identified conversion of pyruvate into acetate by thiamine-dependent keto acid dehydrogenases as well as a ROS-coupled reaction [75].

Acetyl-CoA is converted to malonyl-CoA by acetyl-CoA carboxylase (ACC, encoded by ACACA and ACACB), with both acetyl-CoA (1 molecule) and malonyl-CoA (7 molecules) used by fatty acid synthase (FAS, encoded by FASN) to produce the 16 carbon saturated fatty acid palmitate. The production of one palmitate molecule requires 7 ATP and 14 molecules of NADPH and the molecular regulation of de novo fatty acid synthesis pathway has been comprehensively reviewed [76]. It should be noted that cytosolic acetyl-CoA is also a substrate for cholesterol synthesis (in fact, all 27 carbons in cholesterol are derived from acetyl-CoA [77]), and protein acetylation [78]. In contrast, malonyl-CoA is also a substrate for fatty acid elongation (see below).

Increased de novo fatty acid synthesis is a commonly observed feature of cancer cells [79, 80], and the enzymes ACLY, ACC, and FAS have been demonstrated as potential therapeutic targets. Recent examples include knockdown of ACLY impairing pancreatic tumor formation [81] and knockdown of FASN blocking tumor development in mTOR-driven liver cancer [82], while pharmacological inhibition of FAS reduced tumor growth in preclinical models of castration-resistant prostate cancer [83]. Similar observations have been reported in breast [84] and colon [85] cancer. As such, this is an area of ongoing drug development including the recently developed and characterized selective, irreversible, and potent FASN inhibitor IPI-9119 [83], as well as the recent report of a new mechanism to inhibit human ACLY [86].

De novo fatty acid synthesis has received significant attention; however, first principle questions remain. For example, why is the palmitate produced by de novo fatty acid synthesis required for cell viability? It is widely held that the increase in de novo production of palmitate is to meet demand for membrane synthesis of highly proliferative cancer cells. If this is so, one would assume that de novo synthesis of fatty acids is a greater contributor to bulk lipid synthesis than other pools. We and others have quantified the relative contribution of extracellular fatty acids and de novo synthesis of fatty acids to the cellular lipid pools in cancer cells. De novo synthesis of fatty acids from extracellular glucose contributes ~ 20–30% of cellular lipids, whereas glutamine contributes ~ 5% in H1299 and A549 non-small cell lung cancer cell lines [87], MCF-7 and MDA-231 cells [5] and a range of prostate cancer cell lines [4]; with the remainder (~ 65–75%) coming from extracellular fatty acids. These reports compliment observations made by the Nomura laboratory that showed that de novo synthesized palmitate, generated using extracellular isotopically-labelled glucose, are incorporated into a broad range of lipids including glycerophospholipids, glycerolipids, and sphingolipids but account for only a small fraction of the total levels of palmitate-containing lipids [88]. Specifically, the [^13^C]C16:0 FFA (m+16) pool, which represents newly synthesized palmitate from ^13^C-labelled glucose (incubated for 4 h), accounted for only ≤ 1.9% of the total free palmitate pool in five different cancer cell lines. Likewise, [^13^C]C16:0 was only a minor fraction of the total pool of other lipid pools; [^13^C]lysophosphatidate (m+19) was up to 14% of the total C16:0 lysophosphatidate pool, with similar patterns observed in the C16:0/C18:1 phosphatidate pool, C16:0/C18:1 diacylglycerol pool, C16:0/C18:1 phosphatidylserine, C16:0/C18:1 lysophosphatidylcholine, and C16:0/C18:1 lysophosphatidylethanolamine [88]. In general, de novo synthesized palmitate was not the majority source of C16:0 acyl chains for the broad range of lipids that were measured. Critically, these observations were made using serum-free conditions, and so likely represent the maximal contribution of de novo synthesized fatty acids to membrane synthesis as there were no competing extracellular lipids. The Rabinowitz laboratory also assessed the contribution of de novo palmitate synthesis in cells cultured in 25 mM ^13^C-labelled glucose, 4 mM ^13^C-labelled glutamine, and 10% dialyzed FBS for greater than five doublings (compared to 10 mM glucose, serum-free, 4 h ^13^C-glucose incubation for [88]). They reported that de novo synthesized palmitate generated in cells cultured in labeled glucose and labeled glutamine accounted for ~ 75–90% of the total cellular C16:0 pool, including fatty acyl chains from complex lipids [45]. The authors also reported the incorporation of these labeled non-lipid substrates into C18:0 and C18:1, including m+16 and m+18 isotopologues, indicating that a fraction of de novo synthesized palmitate is modified before being incorporation into lipids (discussed in greater detail in the “Modification of free fatty acids: fatty acid elongation” section). Overall, the majority of reports demonstrate that extracellular fatty acids contribute to the building of lipids to a greater extent than non-lipid substrates in cell culture. This pattern may or may not occur in vivo as it remains unclear what the fatty acid/lipid levels are in the tumor microenvironment, which themselves almost certainly differ in the primary tumor and metastatic tissues as well as at different sites of metastasis.

As de novo synthesized palmitate is not the major source for glycerophospholipid synthesis, it remains unclear why the activity of enzymes that produce palmitate de novo is critical for cell viability. It is conceivable that de novo synthesized palmitate, or its subsequently modified (i.e., elongated, desaturated) variants, are partitioned into specific lipids that are essential for cellular functions. This concept is supported by the observation that FAS inhibition sensitivity correlated with the incorporation of de novo-synthesized palmitate into (C16:0)lysophosphatidate, (C16:0/C18:1)DG, (C16:0/C18:1)PC, and (C16:0)LPC, rather than the free palmitate pool [88]. This suggests that the cell viability in FAS inhibitor sensitive cells is dependent upon the production of specific complex lipids. However, it remains to be determined whether this is also the case for other glycerophospholipids that incorporate elongated and/or desaturated de novo-synthesized palmitate. It is important to note that the inhibition of de novo fatty synthesis is robust primarily in tissue culture conditions where extracellular lipids are depleted, including low serum conditions, serum-free, or delipidated FBS [88–90]. The ever-growing use of lipidomic analyses, in combination with stable isotopes, are likely to provide greater insight into membrane and other lipid pool composition and probe the biological function(s) of de novo-synthesized fatty acids.

One of the other aspects of palmitate metabolism that remains to be resolved, especially in terms of its requirement for cell viability, is the fact that palmitate supplementation of cell culture media leads to lipotoxicity and activation of apoptosis. This is consistently observed in a broad range of cell lines, including 3T3 fibroblasts [91], peripheral blood mononuclear cells [92], macrophages [93], and hepatocytes [94], as well as cancer cells lines [3, 4, 92, 94–101]. We recently demonstrated that the higher rates of fatty acid oxidation in C4-2B prostate cancer cells and MCF-7 breast cancer cells protect from palmitate-induced apoptosis, and inhibition of mitochondrial fatty acid oxidation sensitized these cells and lead to increased cell death [3, 4]. Palmitate induced apoptosis in PC-3 prostate cancer cells and MDA-MB-231 breast cancer cells was prevented by pre-treatment of these cells with FAs (oleate or oleate:palmitate:linoleate mix), and this protective effect required DGAT-1–mediated triacylglycerol synthesis. More recently, palmitate-induced apoptosis was reported to require endoplasmic reticulum glycerol-3-phosphate acyltransferase activity and the formation of di-saturated glycerolipids [102]. Collectively, these observations point toward a scenario whereby intracellular palmitate levels, influenced by intracellular and extracellular sources, are tightly controlled and that insufficient or too much results in cell death.

While we have not discussed in great detail the role of de novo fatty acid synthesis in cancer beyond the relative contribution of palmitate to lipid synthesis, we believe that relative to other areas of tumor fatty acid metabolism, our understanding of this pathway in oncogenesis has not dramatically advanced since the excellent review of this pathway by Röhrig and Schulze [76]. As such, we have prioritized other facets of fatty acid metabolism and their emerging roles that have been reported in recent years.

##### Lipolysis of membrane lipids

**Fig. 2:**
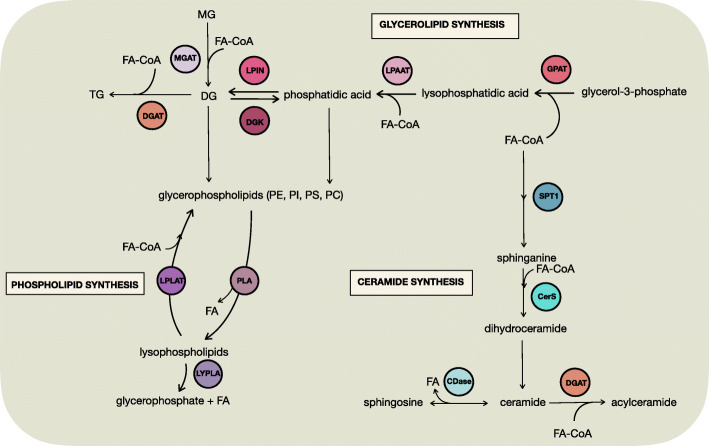
Simple and complex lipid synthesis pathways. Fatty acyl-CoAs are used as building blocks for glycerolipids, glycerophospholipids, and ceramides and are attached to glycerol or sphingosine backbones through actions of acyltransferases and ceramide synthases. Likewise, acylceramides are generated from ceramide and fatty acyl-CoA. Fatty acids can be liberated through the actions of phospholipases, lysophospholipases, and ceramidases. *CDase* ceramidase, *CerS* ceramide synthase, *DG* diacylglycerol, *DGAT* diacylglycerol acyltransferase, *DGK* diacylglycerol kinase, *FA*-*CoA* fatty acyl-CoA, *FA* fatty acid, *GPAT* glycerol-3-phosphate acyltransferases, *LPAAT* lysophosphatidate acyl transferase, *LPLAT* lysophospholipid acyltransferase, *LYPLA* lysophospholipase A, *MG* monoacylglycerol, *MGAT* monoacylglycerol acyltransferase, *PC* phosphatidylcholine, *PE* phosphatidylethanolamine, *PI* phosphatidylinositol, *PLA* phospholipid lipase, *PS* phosphatidylserine, *SPT1* serine palmitoyltransferase 1, *TG* triacylglycerols

Complex membrane lipids are also an input source for the intracellular fatty acid pool. Membranes are not a static cellular structure but constantly undergo remodeling via the removal of fatty acids and the addition of fatty acyl-CoAs to glycerophospholipids via the competing actions of phospholipases and acyltransferases (Fig. 2). Phospholipases vary in their structure and function with the acylhydrolases PLA_1_, PLA_2_, and PLB enzymes liberating free fatty acids from specific sites of the phospholipid (i.e., sn-1 or sn-2) producing lysophospholipids (also called monoacylglycerophospholipids [103])—see the review by Harayama and Riezman [26] for details of the chemical diversity of membrane lipids. Other family members include PLC and PLD that hydrolyze glycerophospholipids, but target head group phosphates and so do not liberate a fatty acid. The H-RAS-like suppressor (HRASLS) subfamily all possess in vitro PLA_1_ and PLA_2_ activities (producing fatty acids) as well as O-acyltransferase activities to remodel glycerophospholipid acyl chains [104]. Likewise, lysophospholipids can also be hydrolyzed to produce glycerophosphate and a fatty acid by the actions of the cytosolic serine hydrolases lysophospholipase A1 (LYPLA1) and lysophospholipase A2 (LYPLA2) [105]. Interestingly, LYPLAs also exhibit protein palmitoyl thioesterase (i.e., depalmitoylation) activity to produce palmitate, with targets including oncogenes HRAS and SRC [106].

A number of studies have explored the role of PLA_1_, PLA_2_, and PLB family of enzymes in cancer cell biology, with most centered on PLA_2_-mediated release of arachidonic acid, which is then used as a substrate for eicosanoid synthesis [107, 108]. That said, the contribution of liberated fatty acids other than arachidonic acid to the free fatty acid pool is not described. In general, PLA_2_ activity, including HRASLS expression, is lower in breast, ovarian, and other cancer cells compared to respective normal cells [104, 109]. Conversely, LYPLA1 plays a tumor-promotor role in non-small cell lung cancer cells [110], which is unlikely to be linked to the liberation of fatty acids from lysophospholipids contributing to the free fatty acid pool, but through changes in lysophospholipid levels which can regulate several signaling pathways including MAPK and ERK [105] or via depalmitoylation of the α-subunit of G-proteins and proto-oncogene H-Ras products [111].

Similar to membrane glycerophospholipids, sphingolipids can be hydrolyzed to release fatty acids. As an example, ceramides are substrates for the ceramidase family of enzymes, which produce a free fatty acid and a sphingosine molecule (Fig. 2) [112]. Ceramidases are classified by their optimal pH for catalytic activity, i.e., acid, neutral, alkaline. A recent review highlighted that acid ceramidases are commonly overexpressed in a range of cancer types [113]. Further, a role for neutral ceramidase in colon cancer biology has been demonstrated [114]; however, the role of the fatty acid that is liberated by ceramidases has not been investigated. This is likely due to the fact that this reaction also produces sphingosine which can be phosphorylated by sphingosine kinases to form sphingosine-1-phosphate, and can activate sphingosine-1-phosphate receptors to influence cancer cell biology (see review [115]).

##### Lipophagy and lipolysis of intracellular lipid droplets

**Fig. 3:**
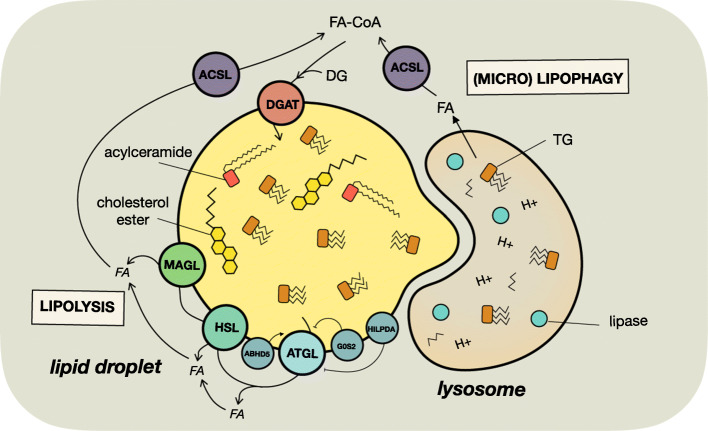
Lipolysis and lipophagy of lipid droplet contained neutral lipids. Neutral lipids including triacylglycerols, cholesterol esters, and acylceramides are broken down through the actions of neutral lipases (lipolysis) or lipophagy to liberate fatty acids. Triacylglycerol lipolysis is catalyzed by a series of reactions by ATGL, HSL, and MAGL. ATGL activity is activated by protein-protein interaction with ABHD5 and suppressed by G0S2 and HILPDA. Fatty acids liberated by lipolysis or lipophagy are activated by ACSL to form fatty acyl-CoAs. Triacylglycerol and acylceramide synthesis are catalyzed by DGAT using fatty acyl-CoA and diacylglycerol or ceramide as substrates. *ABHD5* abhydrolase domain containing 5, *ACSL* long-chain acyl-CoA synthase, *ATGL* adipose triacylglycerol lipase, *DG* diacylglycerol, *DGAT* diacylglycerol acyltransferase, *FA*-*CoA* fatty acyl-CoA, *FA* fatty acid, *G0S2* G0/G1 switch gene 2, *HILPDA* hypoxia-inducible lipid droplet-associated protein, *HSL* hormone-sensitive lipase, *MAGL* monoglyceride lipase

Another intracellular source of fatty acids is neutral lipids stored in cytosolic lipid droplets, including triacylglycerols (TGs; 3 fatty acids attached to a glycerol backbone), sterol esters (1 fatty acid attached to 1 sterol), and 1-O-acylceramides (1 fatty acid attached to 1 ceramide) [116, 117]. The mobilization of lipid droplet-derived fatty acids occurs via the actions of cytosolic neutral lipases or lipophagy [118] (Fig. 3). The molecular regulation of lipolysis of lipid droplet-contained TG is complex and involves a combination of subcellular localization, post-translational modification (in particular phosphorylation), and protein-protein interaction [118]. In contrast, our understanding of the regulatory mechanisms of lipophagy and hydrolysis of sterol esters and 1-O-acylceramides remains underdeveloped.

Lipid droplet accumulation has been reported in a broad range of human cancers (see review [119]), in hypoxic cancer cells [41], and several studies have linked lipid droplet accumulation with more aggressive disease [119–121]. In fact, lipid droplets are key managers of the storage and release of unsaturated fatty acids and play an important antioxidant role and protect cancer cells from stress associated with both nutrient excess and nutrient deprivation [42, 122]. As such, they have been proposed to play direct roles in many of the Hallmarks of Cancer (see review [123]).

The first step is TG hydrolysis, which releases one fatty acid and produces a diacylglycerol (DG). Several enzymes express TG hydrolase activity, including the adipose triglyceride lipase (ATGL, encoded by *PNPLA2*) and hormone-sensitive lipase (HSL, encoded by *LIPE*), as well as carboxylesterase 2 (CES2), alongside its well-described role in drug metabolism [124], and patatin-like phospholipase domain-containing protein 3 (PNPLA3), in human liver [125], but it remains unclear whether CES2 and PNPLA3 participate in TG hydrolysis in cancer cells.

ATGL targets the sn-2 position of TG to produce an sn-1,3 DG, that does not activate protein kinase C signaling (see below). There are many conflicting observations reported regarding the role of ATGL in cancer biology; note that this inconsistency in the role of ATGL is also observed in fatty liver [126, 127]. Some studies have shown that ATGL loss-of-function reduces cancer cell proliferation and invasion (see review [128]), whereas others have reported increased lung cancer cell proliferation and migration [129], or no change in colorectal, melanoma, lung, and liver cancer cell proliferation or in vivo tumor size [130, 131]. ATGL overexpression did suppress melanoma, lung, and liver cancer cell proliferation [131]. We and others have shown that ATGL protein levels, and TG levels, are increased in colon, breast, and prostate cancer cells in response to high levels of extracellular fatty acids [3, 4, 132] and co-culturing with adipocytes [133], which also increase cell proliferation. Importantly, we also showed that the increase in ATGL protein and intracellular TG levels increased the rate of mitochondrial oxidation of TG-derived fatty acids in breast and prostate cancer cells [3, 4]. This suggests that ATGL likely provides fatty acids that are key for mechanisms that support cell proliferation and invasion, and that tumors growing in lipid-rich environments (such as obesity) have enhanced fatty acid flux and thereby increased cell proliferation and invasion rates.

Complicating our understanding of the role of ATGL catalyzed TG hydrolysis in cancer cell biology is the fact that the activity of ATGL is regulated by protein-protein interactions. To date, ABHD5 (also known as CGI-58) is the only identified co-activator of ATGL [134]. Knockdown of ABHD5 reduced the rate of TG hydrolysis and increased TG level as expected in prostate cancer cells, but one study reported activated apoptotic signaling [135], whereas another showed induced epithelial-mesenchymal transition, leading to increased cell invasion and proliferation [136]. Interestingly, a recent study showed that cancer cell TG hydrolase activity in vitro is not activated by ABHD5 [131], while others have reported that ABHD5 possesses lysophosphatidate acyl transferase (LPAAT) activity, converting lysophosphatidate into phosphatidate [137]. As such, the influence of ABHD5 on cancer cell biology, affecting fatty acid mobilization, maybe through ATGL-dependent and independent mechanisms, but the impact on oncogenesis remains to be defined.

Several proteins act as co-suppressors of ATGL, including G0/G1 switch gene 2 (G0S2) [134], and hypoxia-inducible lipid droplet associated protein (HILPDA; also known as hypoxia-inducible gene 2, HIG2) [130, 132]. Knockdown of HILPDA increased lipid droplet-derived fatty acid mobilization, due to less suppression of ATGL activity, resulting in increased fatty acid oxidation and ROS production, and impaired the growth of HCT116 colon carcinoma and HeLa tumor xenograft growth [130]. Similar observations were reported in HILPDA loss-of-function studies in HCT116 colon carcinoma models [132]. Overall, reduced ATGL activity, via knockdown of ATGL or ABHD5, has similar effects on cancer cell proliferation and viability as increased ATGL activity, via overexpression of ATGL or knockdown of HILPDA. This suggests that too much or too little TG hydrolysis and fatty acid mobilization has deleterious effects on cancer cell viability, but it remains unclear by what mechanism(s) this occurs. It is possible that it may involve the prevention of lipotoxicity as well as PPAR-α signaling (see review [128]).

The next step in the lipolytic cascade is the hydrolysis of sn-1,3 DG into MG and the release of one fatty acid (Fig. 3). This reaction is catalyzed by HSL, which has broad substrate specificity including cholesteryl esters, TG, MG, and retinyl esters [138], and to date, little is known about the role of HSL in cancer. The final step in neutral lipolysis is catabolism of MG by monoglyceride lipase (encoded by *MGLL*) to produce a fatty acid and glycerol. Despite the MG pool being small relative to TG, DG, and glycerophospholipids levels, monoglyceride lipase exerts significant influence on cancer cell behavior [139, 140], and MG hydrolysis enzyme activity is more than 11-fold higher in cancerous lung tissues than in paired non-cancerous tissues [141]. Recently, ABHD6 has been reported to be the primary MG lipase in NSCLC and blockade of ABHD6 significantly reduced the migration, invasion, and in vivo tumor growth of NSCLC [141]. Importantly, the loss-of-function of monoglyceride lipase was partly rescued by exogenous palmitate supplementation, thereby demonstrating that the fatty acids liberated by MG hydrolysis are essential mediators of cancer cell migration [140].

Lipid droplets also store sterol ester, in particular cholesteryl ester, and 1-O-acylceramides, which have a fatty acyl attached that is liberated during hydrolysis and contributes to the intracellular fatty acid pool. The molecular mechanisms of cholesteryl ester hydrolysis are overall poorly understood, with many candidate enzymes being proposed to possess cholesteryl ester hydrolase activity. They include CES1, HSL, KIAA1363/NCEH1, and possibly CES3 [142–144], but no consensus has been reached, let alone insight into their role in cancer. It will be challenging to determine the role that these cholesteryl ester hydrolase candidates play in fatty acid metabolism as many of these enzymes have an affinity for multiple lipid and non-lipid substrates (i.e., (pro)drugs and environmental toxicants) and that cholesteryl ester hydrolysis will influence cellular cholesterol levels as well as fatty acid levels [124, 143]. Finally, the molecular regulators of lipid droplet contained 1-O-acylceramide hydrolysis are unknown and so the role that 1-O-acylceramide breakdown in cell biology is yet to be described.

Lipid droplet-contained fatty acids can also be mobilized via lipophagy [145], which is the autophagic degradation of lipid droplets (Fig. 3). Some of the molecular mechanisms that facilitate lipophagy have been identified, and recent examples include RAB7 and ATG5 [146, 147], PNPLA5 [148], ATG14 and Ulk1 [149], MAP1S [150], and LAMP1 [151]. In general, lipophagy plays a tumor suppressor role as it leads to increased intracellular free fatty acid levels, which promotes cell death via ferroptosis, ROS production, and ER stress [149, 151]. However, it has also been proposed that autophagy can play a pro-tumor role in nutrient-deprived situations where mobilization of fatty acids via this pathway is used for subsequent catabolic and anabolic processing [145]. Currently, the contribution of lipid droplet-containing fatty acids to the intracellular fatty acid pool, relative to the other sources, let alone whether these are mobilized by lipolysis or lipophagy, is unknown.

Overall, cancer cells have many diverse sources of fatty acids that can supply the intracellular fatty acid pool. The stoichiometric relationships of the various supply lines remain to be defined. Still, they are likely to be heavily influenced by substrate availability, including low or high extracellular fatty acid and lipoprotein levels, as well as non-lipid precursors for de novo fatty acid synthesis. Further, the relationships between these pathways will also be influenced by extracellular cues, including hormonal stimulation and oxygen availability (see reviews [39, 40]).

### Modification of free fatty acids

In this section, we will focus on a range of reactions that modify intracellular free fatty acids, including activation, desaturation, and elongation. We will not be discussing the role that fatty acid binding proteins (FABPs) play in tumor fatty acid metabolism and cell biology as it likely involves both FABP-mediated events and fatty acid delivery aspects. The diverse roles of FABPs in cancer development and progression were recently reviewed [152].

#### Activation and deactivation

Free fatty acids are biologically toxic to cells but are themselves not substrates for downstream metabolic pathways, with only a very small number of exceptions (i.e., eicosanoid synthesis). Irrespective of where free fatty acids are derived, they must first be activated by esterification with CoA to form fatty acyl-CoAs using two high-energy bonds from ATP [153] (Fig. 1). This activation step is catalyzed by acyl-CoA synthetases (ACSs), which consists of sub-families determined by the acyl chain length: short-chain ACSs, medium-chain ACSs, long-chain ASCs (ASCLs), and very long-chain ACSs. Fatty acid transport proteins (FATPs, members of the Slc27 family) also have acyl-CoA synthetase activity, which is likely to be the mode of action for how these proteins influence the uptake of extracellular fatty acids [154]. The sub-families of ACSs have multiple isoforms; for example, there are five isoforms of ACSL expressed in mammalian cells that have defined substrate specificity [155]. Further, fatty acid activation is highly compartmentalized due to subcellular localization of ACS family members. An example is the non-overlapping intracellular distribution of ACSL3 and ACSL4 in HT1080 and MCF-7 breast cancer cell lines [156]. This complexity likely explains the lack of a consistent or straightforward relationship between the levels of the five ACSL family members and cancer; with some family members having increased protein levels and expression, whereas other family members having decreased levels [155]. For example, ACSL4 has been regularly reported to be overexpressed in multiple cancer types but downregulated in others [155], whereas high expression of ACSLs 1, 3, and 5 associate with a favorable prognosis in patients with lung cancer [157]. It is also conceivable that the altered fatty acyl-species profile of cancer cells and tumor (i.e., altered MUFA/PUFA ratio; see below) drives a change in ACSL expression and localization. An interesting advance was the identification that the transmembrane glycoprotein, CUB-domain containing protein 1 (CDCP1), a driver of migration and invasion in multiple forms of carcinoma, interacts with many members of the ASCL family in breast cancer, and loss-of-function of CDCP1 increases ASCL activity and lipid droplet abundance and reduces fatty acid oxidation and impairs cell migration [158].

Fatty acyl-CoAs can be hydrolyzed via the actions of acyl-CoA thioesterases (ACOTs) to produce a free fatty acid and CoA-SH [159] (Fig. 1). Like ACSs, ACOT family members differ in their subcellular localization, including localizing within the cytosol, peroxisomes, endoplasmic reticulum, and mitochondria, as well as substrate specificity (see review [160]). While the majority of fatty acid metabolic pathways use fatty acyl-CoAs as substrates (discussed next), a key exception is arachidonic acid which is the substrate for eicosanoid synthesis, not arachidonoyl-CoA [161]; with arachidonoyl-CoA a substrate for ACOT7 [162]. The levels of ACOTs are altered in tumors; for example, increased expression of ACOT1 correlates with clinicopathological parameters and poor prognosis in gastric adenocarcinoma [163], and ACOT11 and ACOT13 are increased in clinical specimens of lung adenocarcinoma [164]. Likewise, high expression of ACOTs (7, 11, and 13) was associated with poor prognosis in patients with lung cancer, but interestingly, high expression of ACSLs (1, 3, and 5) associates with a favorable prognosis [157]. Functionally, pharmacological inhibition of ACOT activity and genetic loss-of-function of ACOT7 induced cell cycle arrest and reduced cell growth in breast and lung carcinoma cells [161]. These changes in ACOT expression reported in clinical cancer tissues [157, 161, 163, 164] would be predicted to change the tumor lipidome and thereby behavior. However, the effect of ACOT on the tumor lipidome has not been reported but data from non-cancer tissues points to very subtle changes. Specifically, overexpression of ACOT7 in mouse macrophages had only mild effects on glycerophospholipid levels; specifically, subtle increases in phosphatidylcholine (PC) and phosphatidylethanolamine (PE) saturated fatty acyl species and reductions in MUFA species [165], whereas loss-of-function of ACOT7 nominally increased the abundance of glycerophospholipids containing unsaturated acyl-chains, but importantly not arachidonic acid-containing glycerophospholipid species [166]. As such, the precise mechanism by which these ACOT isoforms influence cancer cell behavior is unknown. It is likely linked to the balance between fatty acyl-CoAs and free fatty acid levels, which themselves are influenced not only by the ratio of ACOT and ACS(L) protein levels (and thereby thioesterase and acyl CoA synthetase activity) but also the subcellular localization of these reactions, to influence the partitioning of fatty acids/fatty acyl-CoAs. To date, there is little knowledge of this balance in cancer, but unsurprisingly, the basal in vitro ACS activity is much higher than thioesterase activity in mouse skeletal muscle [165], as such there is a bias toward acyl-CoA synthesis compared to acyl-CoA hydrolysis. The complex role of ACOTs and ACSLs play in influencing tumor fatty acid metabolism remains poorly defined.

Fatty acyl-CoAs can be modified prior to esterification or oxidation (Fig. 1). The two main modifications are desaturation and elongation. Both modifications impose significant biophysical changes to both the free fatty acid (following de-activation/removal of CoA) as well as the complex lipids that contain these modified fatty acyl chains.

#### Fatty acid desaturation

The introduction of a double-bond between carbons of fatty acyl-CoAs is performed by the actions of desaturases, which use NAD(P)H and O_2_ as co-factors (Fig. 1) [167]. Desaturases introduce double bonds in a chemo-, regio-, and stereoselective manner [168]. One of the most well-studied desaturases is the delta-9 desaturase stearoyl-CoA desaturase (SCD), which despite its name, has substrate specificity for saturated fatty acyl-CoAs of 12 to 19 carbons, including palmitoyl-CoA and stearyl-CoA [169]. Mammalian cells do express other desaturases that predominantly produce polyunsaturated fatty acids, including delta-5 (encoded by FADS1) and delta-6 (encoded by FADS2) fatty acid desaturases, as well as FADS3 whose gene product has been reported to modulate docosahexaenoic acid (DHA, 22:6n-3) levels in liver and brain [170], acts as a Δ14Z sphingoid base desaturase [171], and catalyzes trans-vaccenate Δ13-desaturation [172]. Mammalian cells do not express delta-12 and delta-15 desaturases, which explains why linoleic acid (18:2^Δ9,12^) and linolenic acid (18:3^Δ9,12,15^) are essential fatty acids.

SCD catalyzes the biosynthesis of monounsaturated fatty acids, and its role in cancer has been previously reviewed [173, 174]. In the years since those reviews were published, increased expression of SCD has been reported in an ever-growing list of cancer types including breast [57], colorectal [175], ovarian [176], endometrial [177], bladder [178], colorectal cancer [175], and clear-cell renal cell carcinoma [179]. Many of these recent studies have demonstrated that inhibiting SCD leads to accumulation of palmitate and stearate saturated fatty acids and reduced palmitoleate and oleate monounsaturated fatty acids [175, 176, 180, 181]. These studies also report reduced cell proliferation and migration, increased ceramide synthesis, and activated apoptosis and ferroptosis [57, 175–178, 180, 182]. However, deletion of SCD in the intestinal epithelium of mice resulted in more and larger tumors [181]. Despite this apparent difference in the role of SCD in tumorigenesis in colon cancer and disease progression in other cancer types, inhibition of SCD activity can be rescued by oleate supplementation [57, 175, 176, 181–183], but not palmitate [183]. Interestingly, the accumulation of palmitate during SCD inhibition stimulated de novo ceramide synthesis, which activates apoptosis in colorectal cancer cells [183]. The same authors demonstrated that inhibition of de novo ceramide synthesis reversed the tumor shrinkage that arose from SCD inhibition. The overall view is that SCD plays a role in mitogenic and stress-related signal transduction pathways, but it remains to be determined whether lipid factors, such as altered saturated/MUFA profiles, mediate the pleiotropic activities of SCD in cancer cell biology (see review [184]).

Alongside these recent advances in the understanding of SCD biology and monounsaturated fatty acid production in cancer, an alternative desaturation pathway was recently identified in hepatocellular carcinoma and non-small cell lung cancer [180]. These specific cancer types are insensitive to pharmacological inhibition of SCD as they upregulate delta-6 desaturase (FADS2) to produce the monounsaturated fatty acyl-CoA sapienyl-CoA (C16:1, n-10) instead of palmitoleoyl-CoA (C16:1, n-9) from palmitoyl-CoA. As such, this maintains monounsaturated fatty acid levels to avoid the accumulation of saturated fatty acids and an imbalance between MUFA and PUFA levels (discussed below).

Delta-6 desaturase (FADS2) also works in series with delta-5 desaturase (FADS1) in the synthesis of the PUFA arachidonoyl-CoA (20:4^Δ5,8,11,14^) and docosahexaenoyl-CoA (22:6^Δ4,7,10,13,16,19^) from linoleoyl-CoA (18:2^Δ9,12^) and linolenoyl-CoA (18:3^Δ9,12,15^) respectively [185]. These other members of the desaturase family have received little recent attention from the cancer biology field compared to SCD (see review [174]). Breast tumors and breast cancer cell lines have reduced levels of delta-6 desaturase (FADS2) compared to non-malignant cells [186, 187], as is delta-5 desaturase (FADS1) in non-small-cell lung cancer [188] and esophageal squamous cell carcinoma [189]. Interestingly, FADS2 overexpression in MCF-7 breast cancer cells, which have no detectable basal Δ6-desaturase activity, increased the endogenous biosynthesis of the polyunsaturated fatty acids docosahexaenoic acid (22:6n-3) and docosapentaenoic acid (22:5n-6) via a delta-4 desaturation reaction, to add to the well-established delta-6 and delta-8 desaturation activity of the FADS2 gene product [190]. To some extent, the low levels of PUFA synthesizing enzymes in cancer cells is reflected in relatively lower levels of many PUFA species, compared to MUFA (discussed below in detail). Further, this altered MUFA/PUFA ratio is advantageous for cancer cells as it results in fewer peroxidation susceptible targets and reduced susceptibility to ferroptosis (i.e., iron-dependent cell death) [191]. Recent studies have provided new insights into the mechanisms that regulate ferroptosis, including the requirement for acyl-CoA synthetase activity [191, 192] and the enrichment of PUFA in ether phospholipids [193] and PEs [194]. These studies and others (see review [195]) provide advances in our understanding of the mechanisms that regulate ferroptosis; however, the precise role that PUFA synthesis, that is catalyzed by delta-6 and delta-5 desaturases, in ferroptosis activation and cell death in cancer remains to be defined.

#### Fatty acid elongation

Endogenously synthesized and exogenously-sourced fatty acids can be progressively extended in length (i.e., elongated) by two-carbon units after they have been activated as fatty acyl-CoAs (Fig. 1) [196]. Malonyl-CoA is the source of the additional carbons which is added to long-chain fatty acyl-CoAs by a series of reactions catalyzed by the elongation of very long-chain fatty acid enzymes (ELOVL1–7), 3-ketoacyl-CoA reductase (KAR; also known as 17β-hydroxysteroid dehydrogenase type 12, 17β-HSD12 or SDR12C1), 3-hydroxyacyl-CoA dehydratases (HACD1–4), and 2,3-trans-enoyl-CoA reductase (TER). This is followed by two reduction reactions using two NADPHs as co-factors and one dehydration reaction. ELOVLs catalyze the rate-limiting step in the elongation reaction and the seven members of the enzyme family exhibit characteristic substrate specificities toward fatty acyl-CoAs and in their tissue distribution [196–198]. Membrane lipid elongation and/or enhanced ELOVL expression is a common feature in cancer when compared to matched normal tissue [199, 200] and, as targeting ELOVLs is efficacious in cancer models [201–204], membrane lipid elongation appears to promote cancer progression. For example, ELOVL2 activity increases membrane long-chain PUFA content in order to promote epidermal growth factor receptor (EGFR) signaling through membrane domains [205]. Intriguingly, there is also evidence that ELOVL-mediated elongation of fatty acids can impact cancer cell biology beyond their effects on membrane composition and packing. Mutation of p53 in pancreatic cancer cell lines reduced acyl chain lengths of PI-based glycerophospholipids, but had no effect on chain length in PC species which, like PI, are derived from the same precursor, phosphatidate [206]. As PI lipids form the scaffold for PI3K signaling at the plasma membrane, it is possible that specific oncogenic alterations may act via regulating the production of the second messengers that control cancer cell growth and survival. In prostate cancer, knockdown of ELOVL7 reduced saturated fatty acids in membrane glycerophospholipids but also reduced the levels of cholesterol, the critical precursor of the androgen hormones that drive prostate cancer growth [203]. By producing arachidonic acid, elongation of omega-6 PUFAs is essential for the generation of inflammatory and signaling eicosanoids [207], and also generates NAD+, which sustains glycolysis [208].

Most attention has centered on elongation and desaturation of de novo-synthesized fatty acids; however, it is critical to acknowledge that exogenous fatty acids are also substrates for these reactions. This was elegantly demonstrated by Robert and colleagues where radio-labelled palmitate (C16:0) was detected in the C16:1 and C18:1 fatty acyl-chains of glycerophospholipids at greater rates in two glioma cell lines than normal astroblasts [209]. Similarly, radio-labelled stearate (C18:0) was incorporated into the C18:1, C20:1 and C20:3 pools, as was radio-labelled extracellular linoleic acid (C18:2, n-6) and linolenic acid (C18:3, n-3) into other fatty acyl-chains of glycerophospholipids. Most strikingly was the observation that extracellular oleate (C18:1) was not modified into other fatty acyl species of membrane lipids. Similar observations were reported in HepG2 human hepatoma cells using stable isotope labelling and mass spectrometry, where extracellular stearate (C18:0) was the source for 88% of arachidonate (C20:0) and 67% of oleate (C18:1) [210]. Collectively, these studies demonstrate that extracellular fatty acids are substrates for elongation and desaturation reactions in cells, not just endogenously sourced fatty acids, and that this capability is enhanced in cancer cells.

### Outputs of the intracellular fatty acyl-CoA pool and their influence on cancer cell behavior

**Fig. 4:**
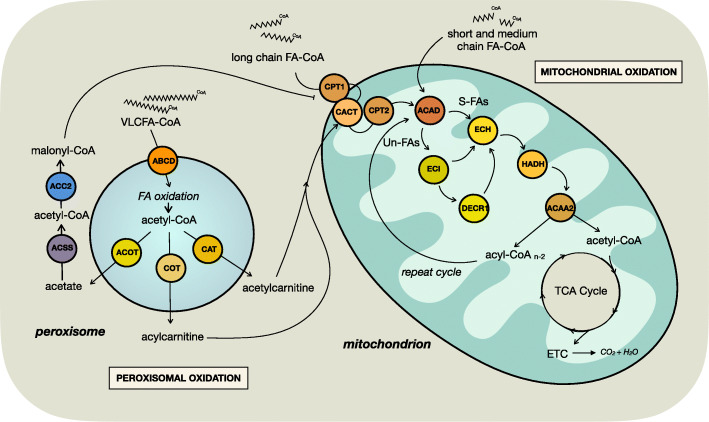
Peroxisomal and mitochondrial fatty acid oxidation. Short- and medium-chain fatty acyl-CoAs freely diffuse into the mitochondria and enter beta oxidation, whereas long-chain fatty acyl-CoAs are transported into the mitochondria via the CPT system. Saturated fatty acyl-CoAs directly enter the beta oxidation pathways, whereas unsaturated fatty acyl-CoAs switch between beta oxidation and the auxiliary pathways which process the double bonds. Beta oxidation shortens fatty acyl-CoAs by two carbons to produce acetyl-CoA which is a substrate for the TCA cycle and ATP generation. Very-long chain fatty acyl-CoAs are transported into peroxisomes via ABCD transporters and undergo oxidation to shorten the fatty acyls and produce acyl-carnitines by carnitine octanoyltransferase which are transported to the mitochondrial, where they are converted to fatty acyl-CoAs by the actions of CPT2. Peroxisomal oxidation also produces acetyl-CoA that can be converted to acetylcarnitine by carnitine acetyltransferase or to acetate by acyl-CoA thioesterases. Mitochondrial fatty acid oxidation is reduced by allosteric inhibition of CPT1 by malonyl-CoA which is produced via ACC2 from acetyl-CoA, which itself generated by acetate by ACSS. *ABCD* ATP-binding cassette transporters, *ACAA2* 3-ketoacyl-CoA thiolase, *ACAD* acyl-CoA dehydrogenase, *ACC2* acetyl-CoA carboxylases, *ACOT* acyl-CoA thioesterases, *ACSS2* cytoplasmic acetyl-CoA synthetase, *CACT* carnitine acylcarnitine translocase, *CAT* carnitine acetyltransferase, *COT* carnitine octanoyltransferase, *CPT1* carnitine palmitoyltransferase 1, *CPT2* carnitine palmitoyltransferase 2, *ETC* electron transport chain, *ECH* enoyl-CoA hydratase, *ECI* Δ3, Δ2-enoyl-CoA isomerase, *DECR1* 2,4-dienoyl CoA-reductase, *HADH* hydroxyacyl-CoA dehydrogenase, *FA*-*CoA* fatty acyl-CoA, *S*-*FAs* saturated fatty acids, *Un*-*FAs* unsaturated fatty acids including MUFAs and PUFAs, *VLCFA*-*CoA* very-long chain fatty acyl-CoA

Fatty acyl-CoAs are substrates for many metabolic pathways, including synthesis of complex lipids, such as glycerolipids and glycerophospholipids (Fig. 2), and generation of energy via β-oxidation (Fig. 4). The coordination of fatty acyl-CoA distribution within the cells has been proposed to involve, in part, acyl-CoA-binding domain-containing proteins (ACBDs) [211]. There are seven family members, including ACBD1, also known as acyl-CoA binding protein, yet little is known about the specific roles of the ACBDs in the regulation of fatty acyl-CoA metabolizing processes [212]. Recently, it was reported that ACBD1 expression is increased glioblastoma multiforme and controls tumor growth by regulating the availability of fatty acyl-CoAs for fatty acid oxidation [213]. If and how ACBDs influence fatty acyl-CoA metabolism in cancer and non-cancerous cells is yet to be defined.

#### Simple and complex lipid synthesis

Fatty acyl-CoAs are the building blocks for the synthesis of glycerolipids (TG, DG), glycerophospholipids (PC, PE, PI), sphingolipids, and sterol esters as key examples [214]. In general, the lipid composition of mammalian cells predominantly consists of PC (~ 45–55%), PE (~ 15–25%), cholesterol (10–20%), PI (10–15%), phosphatidylserine (5–10%), and sphingomyelin (5–10%) [215]. These lipids are distributed heterogeneously within the cell, with organelles possessing unique lipidomes, for example, lipid droplets are rich in TGs, whereas mitochondria uniquely harbor cardiolipin (see review [216]). In general terms, the abundance of lipid classes is altered in cancer when compared to non-cancerous tissue, with PtdIns(3,4,5)P3 and PE levels elevated in cancer [216] while ccRCC lipid droplet rich tumors are defined by increased TG and cholesteryl ester levels but also reduced PE levels [217], and others have reported tumor specific abundance of lysophospholipids and other lipid species (recently reviewed extensively [218]). However, there is great heterogeneity in the tumor lipidome between cancer types [218] and the wide-spread utilization of sophisticated mass spectrometry-based lipidomic applications, alongside mass spectrometry imaging and other spatial approaches, will provide the platform to further define the tumor lipidome.

In this section, we will summarize the synthetic pathways in simple terms while trying to capture the complexity of the system and will avoid in-depth discussions of protein isoforms, subcellular localization, and hormonal regulation.

The synthesis of glycerolipids and glycerophospholipids starts with the acylation of glycerol-3-phosphate, which is derived from glycolysis, at the sn-1 position to form lysophosphatidate via the actions of glycerol-3-phosphate acyltransferases (GPAT) family of enzymes [219] (Fig. 2). GPAT activity is approximately five times faster than fatty acyl-CoA thioesterase activity in mouse skeletal muscle [165, 220], therefore outcompetes fatty acyl-CoA hydrolysis by ACOTs (see above). A second fatty acyl-CoA is attached to the sn-2 position of lysophosphatidate by LPAATs (formerly acylglycerol-3-phosphate acyltransferases (AGPAT)) to produce phosphatidate [221, 222]. Phosphatidate can also be generated from the phosphorylation of DG via the actions of diacylglycerol kinases (DGK) [223].

Phosphatidate is a substrate for several reactions. These include CDP-DG synthases, which replaces the phosphate of phosphatidate with CDP to produce CDP-DG, which itself is a substrate for both PI and phosphatidylglycerol (PG) synthesis. Phosphatidate is also a substrate for cardiolipin synthesis [222]. Finally, phosphatidate is a substrate for lipin phosphatidate phosphatases which de-phosphorylate phosphatidate to produce sn-1,2 DG [222]. sn-1,2 DG can also be generated from MG and a fatty acyl-CoA by the actions of monoacylglycerol acyltransferases (MOGATs). DG is a precursor for several glycerophospholipid classes, including PC, PS, and PE, that is synthesized by a complex array of metabolic reactions which was comprehensively reviewed recently [215]. DG can also be acylated with a third and final fatty acyl-CoA on the sn-3 position to produce triacylglycerol by diacylglycerol acyltransferases (DGAT; Fig. 2). The synthesis of triacylglycerols is a prerequisite for lipid droplet synthesis [224], a process that is highly regulated and complex (see review [225]).

Many intermediates of glycerolipid and glycerophospholipid synthesis act as signaling molecules or have “bioactive” properties. For example, sn-1,2 DG activates protein kinase C signaling, but not sn-1,3 DG, which is produced by ATGL-catalyzed TG hydrolysis (see review [226]). Likewise, phosphatidate regulating mTOR signaling [29–31] and lysophosphatidate acts extracellularly to activate the lysophosphatidate receptor family (see review [227]). These known downstream effects of these bioactive lipids can arise from alterations of multiple enzymes that reside at different subcellular locations, i.e., endoplasmic reticulum versus plasma membrane versus lipid droplet.

Next, we will take a simple biochemical approach, focusing on synthesis and utilization, to discuss the influence of intermediates and end products of glycerolipid and glycerophospholipid synthesis on cancer cell biology. Our approach is based upon the assumption that changes in gene/protein levels will result in changes in lipid levels and thereby affect cell biology. We have attempted to digest this into an easy to follow narrative, but it is undoubtedly a challenging and complex area of cell biology.

The first intermediate is lysophosphatidate which is regulated by GPAT and LPAAT enzymes. Lysophosphatidate levels are lower in human colorectal cancer tissues relative to those in paracarcinoma tissues, which was associated with increased mRNA levels of LPAATγ (AGPAT3) and LPAATδ (AGPAT4) [228]. The lower levels of lysophosphatidate may be due to increased efflux of lysophosphatidate from cancer tissue and thereby act in a paracrine fashion to influence local immune cell function [228]. This would suggest that reduced lysophosphatidate levels promote cancer cell promotion. However, increased GPAT expression, which would be predicted to increase lysophosphatidate levels, is observed in melanoma, lung, prostate, and breast cancer and is associated with shorter overall survival in ovarian cancer and shorter disease-free survival in HER2-positive breast cancer [229]. In fact, knockdown of GPAT1 in breast and ovarian cancer cells, which reduced lysophosphatidate levels, slowed cell growth and migration and was rescued by lysophosphatidate supplementation [230]. As such, it is conceivable that increased GPAT levels promote lysophosphatidate synthesis but at a lesser rate than LPAAT catalyzed conversion of lysophosphatidate into phosphatidate or that the rate of efflux is greater, resulting in reduced lysophosphatidate levels.

The next intermediate is phosphatidate, which is regulated by LPAAT, LPIN, and DGK enzymes as well as PLD (see review [231]), which governs a range of signaling pathways [29–32]. The increased levels of LPAAT in colorectal cancer [228] would be expected to increase the conversion of lysophosphatidate to phosphatidate. However, LPIN1, one of three members of the LPIN family, is highly expressed in ovarian cancer [232], hepatocellular carcinoma [233], and breast cancer [234, 235], and therefore causing an increased conversion of phosphatidate to DG and resulting in no accumulation of PA. Knockdown of LPIN1 reduced incorporation of extracellular palmitate into glycerophospholipids, indicating reduced synthesis and remodeling, which resulted in impaired basal-like triple-negative breast cancer cell viability and orthotopic xenograft growth [234]. This suggests that enhanced conversion of phosphatidate into DG would be advantageous. However, increased levels of DGKs are commonly observed [236–238], which predicts an increased conversion of DG to phosphatidate. In fact, overexpression of DGKα, one of ten isoforms, enhanced cancer cell proliferation and tumor growth, whereas knockdown of DGKα reduced cell viability in a range of cancer types [236–238]. There is some conjecture on the role of or DGKζ, with one study reporting that the levels of DGKζ is elevated in glioblastoma and loss-of-function reduced proliferation [239, 240], whereas DGKζ has been reported as downregulated in HCC and correlated with poorer overall survival [241]. Likewise, DGKγ levels are reduced in colorectal cancer but loss-of-function impaired cell proliferation and invasion [242]. Overall, it is not clear what the consensus view is of phosphatidate levels in cancer cells, or the levels of the various enzymes that regulate its levels.

The final lipid we will discuss in the glycero(phospho)lipid synthesis pathway is DG, which is regulated by LPIN, DGK, and DGAT enzymes, as well as PLCs which de-phosphorylate glycerophospholipids (see review [243]). The reported increased expression of DGK in cancer cells should cause a reduction in DG levels [236–238]; the increase in LPIN levels predicts an increase in DG [232–235]. To complicate our understanding of DG metabolism in cancer, both DGAT isoforms, DGAT1 and DGAT2 that encoded by genes that belong to two distinct gene families [244], are highly expressed in a range of cancers and is associated with increased TG levels and lipid droplet abundance [245, 246]. We recently showed that pharmacological inhibition of DGAT1 in breast and prostate cancer cells blunted TG synthesis and influenced cell viability [3, 4]. Likewise, knockdown of DGAT1 reduced lipid droplet number and cell proliferation and invasion of prostate cancer cells [135, 247] and glioblastoma [246]. However, the protein levels of DGAT2 are reduced in HCC, and overexpression of DGAT2 inhibits cell proliferation and colony formation in vitro and tumor formation in vivo [248]. Both DGAT1 and DGAT2 catalyze the conversion of DG into TG, but they do have distinct and overlapping functions in other cell types [249]. Overall, the role of DG, and other lipid intermediates of the glycero(phospho)lipid synthetic pathway, on cancer cell biology remains to be resolved.

Fatty acyl-CoAs are also building blocks for sphingolipids such as ceramide (Fig. 2). De novo sphingolipid synthesis starts with the condensation of palmitoyl-CoA and serine via the actions of serine palmitoyl-CoA transferase to form 3-ketosphinanine [250]. Following the conversion of 3-ketosphinanine to sphinganine, a fatty acyl-CoA is attached to the backbone by ceramide synthase to produce dihydro-ceramide, which can be further modified to form ceramide and into other complex sphingolipids like sphingomyelin, sphingosine-1-phosphate, and glycosphingolipids [251]. In general terms, ceramide and sphingosine-1-phosphate have opposing roles in regulating cancer cell death and survival, and the role that ceramide synthases and sphingosine kinases have been recently reviewed in detail [115]. Further, we point readers to recent reviews on sphingomyelins and other sphingolipids in cancer [252] as they fall outside the scope of this review.

Another destination for fatty acyl-CoAs is sterol esters, in particular cholesteryl ester, which is the product of the addition of a fatty acyl-CoA to cholesterol that is catalyzed by sterol O-acyltransferases (SOATs), also called acyl-CoA:cholesterol acyltransferases (ACATs). Accumulation of cholesteryl ester in lipid droplets has been reported in pancreatic [253] and prostate cancer [120] as recent examples, and that inhibiting SOAT1 blocked cholesteryl ester synthesis and suppress tumor growth or cancer cell proliferation. It is important to note, as we have previously, that it is challenging to interpret loss-of-function studies of SOATs since altering this reaction will influence both cholesterol and fatty acid levels [120, 254]. That said, a recent study demonstrated an interdependency between the de novo production of oleoyl-CoA via SCD and cholesteryl ester synthesis, at the expense of triacylglycerol synthesis [255]. This suggests that, in certain settings, fatty acyl-CoA availability, in particular oleoyl-CoA, has wide-ranging influences on many aspects of cellular lipid metabolism beyond just glycero- and glycerophospholipid synthesis.

Finally, alongside their contribution to the synthesis of glycerophospholipids, fatty acyl-CoAs are also substrates for cellular membrane remodeling. This remodeling involves the deacylation and acylation of glycerophospholipids, which is called the Lands’ cycle [256]. As highlighted above, PLAs can hydrolyze glycerophospholipids to remove a free fatty acid and produce a lysophospholipid. A new fatty acyl-CoA can be attached to the lysophospholipid by lysophospholipid acyltransferase family of enzymes (LPLAT). This family consists of two subfamilies, the 1-acylglycerol-3-phosphate O-acyltransferase (AGPAT) family and the membrane-bound O-acyltransferases (MBOAT) family [257]. This LPLAT-catalyzed reaction does not alter the abundance of glycerophospholipids (i.e., PC, PE, PS, etc.) but does alter the species based upon the makeup of the fatty acyl chains, i.e., changing the saturation and/or chain lengths of the fatty acyl chains. Several members of the LPLAT family have been linked with cancer cell behavior. For example, elevated lysophosphatidylcholine acyltransferase 1 (LPCAT1) levels are linked with poor prognosis and early tumor recurrence in breast cancer [258, 259], gastric and colorectal cancer [260, 261], prostate cancer [262, 263], ccRCC [264], liver cancer [265], and EGFR-dependent glioblastoma [266]. Tumor tissues and cancer cells with high LPCAT1 expression had increased PC and decreased LPC levels [260, 264, 266], and loss-of-function impaired cell growth and survival [264, 266]. Other members of the LPLAT family have also been implicated in tumor biology, including increased LPCAT2 supporting chemoresistance in colorectal cancer [267, 268], increased protein levels in breast and cervical cancer tissue [269], loss of LPCAT3 enhancing intestinal tumor formation via a cholesterol synthesis mechanism [270], and lysophosphatidylinositol-acyltransferase 1 (LPIAT1) mediated prostaglandin production and non-small cell lung cancer cell growth [271]. It is important to highlight that members of the LPLAT family have substrate specificity in terms of lysophospholipid class (i.e., PC, PI, etc.) and fatty acyl-CoA species which influences the biophysical properties of cell membranes.

#### Protein acylation

Fatty acyl-CoAs are substrates for post-translational attachment to proteins, termed protein acylation or lipidation (Fig. 1). In general, the key examples of protein acylation include *S*-palmitoylation, *N*-palmitoylation, *O*-palmiteoylation, and *N*-myristoylation which use palmitoyl-CoA (C16:0), palmitoleoyl-CoA (C16:1n-9), and myristoyl-CoA (C14:0) as substrates; other fatty acyl-CoAs such as octanoyl-CoA (C8:0) and stearyl-CoA (C18:0) are common medium and long-chain protein acylation substrates (see reviews [272, 273]). A range of enzymes catalyze the addition or removal of fatty acylation post-translational modification of cysteine, serine, lysine or threonine residues, and include DHHC family of protein acyltransferases, Hedgehog acyltransferase, Porcupine, and *N*-myristoyltransferases 1 and 2, and acyl protein thioesterases 1 and 2 [24, 273]. Protein acylation regulates multiple cellular processes including membrane targeting, protein-protein interactions, and intercellular and intracellular signaling, including the regulation of oncogenic Wnt, Ras, and Hedgehog signaling [273], as well as mitochondrial biology [274]. Inhibition of protein acylation has been shown to be a potential therapeutic strategy for many cancers; for example, small molecules that inhibit the acyltransferase Porcupine and thereby *O*-palmiteoylation of Wnt are efficacious in Wnt-dependent cancers [24]. Additional insight into the role of protein acylation in cancer and its therapeutic potential is detailed in recent reviews [272, 273, 275].

#### Mitochondrial and peroxisome oxidation

In principle, the primary catabolic pathway for fatty acyl-CoAs is β-oxidation. Like many other aspects of fatty acid metabolism, specific pathways exist to deal with the diversity of fatty acid species as determined by chain length and desaturation, which we will discuss in some detail.

Increased fatty acid oxidation rates have been reported in many cancer types including lung, breast, liver (see review [276]), and prostate [4]. Further, we recently showed that “receptor-positive” breast and prostate cancer cells (MCF-7 and C4-2B cells respectively) have faster rates of fatty acid oxidation than “receptor-negative” cells (MDA-MB-231 and PC-3 cells) [3, 4], whereas others have reported that triple-negative breast cancer cells with high MYC expressed have faster rates of fatty acid oxidation compared to low MYC expressing triple-negative breast cancer cells and receptor-positive cells (T47D) [277]. Further, these basal rates are increased in a range of cancer cell lines following exposure to high levels of extracellular fatty acids [3, 5] and co-culturing with adipocytes [5, 133].

The rate of fatty acid oxidation is controlled by several mechanisms, including enzyme/protein levels, allosteric regulation of enzyme activity, and substrate availability. Long-chain fatty acyl-CoAs require the carnitine palmitoyltransferase (CPT) system to be shuttled into the mitochondrial matrix (Fig. 4) [76], unlike short- and medium-chain fatty acyl-CoAs that can freely diffuse through the mitochondrial membranes [278]. Readers are pointed to a recent comprehensive review of the metabolism of short- and medium-chain fatty acyl-CoAs for additional insight into their role in cell biology [278]. Briefly, the CPT system consists of CPT1, carnitine-acylcarnitine translocase (CACT) and CPT2, whereby fatty acyl-CoAs are converted to fatty acyl-carnitines by the action of CPT1 on the outer mitochondrial membrane (Fig. 4). CACT is located on the inner mitochondrial membrane and shuttles acylcarnitines into the mitochondrial matrix, where they are reconverted back to fatty acyl-CoA by CPT2 on the matrix side of the inner membrane. CPT1 levels are increased in many cancers and targeting CPT1 impairs cancer cell growth and viability (see reviews [276, 279]). However, others have reported that fatty acid oxidation genes are downregulated in multiple tumor types [280], including clear cell renal cell carcinoma where decreased CPT1 protein levels reduces fatty acid transport into the mitochondria, leading to fatty acid storage in lipid droplets, which is a hallmark feature of ccRCC [281]. Another study reported that increasing CPT1 protein levels in MDA-MB-231 breast cancer cells impaired proliferation and migration [280]. Conversely, others have reported that CPT1 expression is elevated in triple-negative breast cancer cells that overexpress c-Myc, leading to increased fatty acid oxidation, and that inhibition of CPT1 reduced growth of Myc-driven triple-negative breast cancer tumors [277]. Like other aspects of cellular fatty acid metabolism, CPT1 protein levels are increased in response to high levels of extracellular fatty acids [4, 5] and co-culturing with adipocytes [5, 133, 282], associated with an increase in the rate of fatty acid oxidation.

The enzymatic activity of CPT1 is allosterically inhibited by malonyl-CoA, which is produced from acetyl-CoA by ACC. The reverse reaction is catalyzed by malonyl-CoA decarboxylase. Studies in the liver and skeletal muscle have shown that ACC2 is the major isoform that produces malonyl-CoA that inhibits CPT1 as it localizes to the outer mitochondrial membrane [283]; cytosolic ACC1 participates in de novo fatty acid synthesis (discussed above). Protein levels of ACC2 are reduced in a range of acidic pH-adapted cancer cells [284] and during breast cancer cell EMT [285], and associates with increased fatty acid oxidation.

Several mechanisms have been proposed to explain how the inhibition of CPT1 activity, to reduce fatty acid oxidation, slows cell proliferation. These include reduced production of ATP and NADPH levels, which are required for biomass synthesis and redox balance [279, 286]. More recently, it has been shown that inhibition of CPT1 and fatty acid oxidation reduces the activation of the proto-oncogene SRC, including mitochondrially-localized SRC, to result in reduced in vitro and in vivo triple-negative breast cancer cell and tumor growth [287]. Notably, the autophosphorylation of SRC, which is required for its activation, utilize ATP generated from mitochondrial oxidative phosphorylation, and in turn activated Src phosphorylates mitochondrial ETC proteins to maintain its activated status, and thereby regulate mitochondrial function and cell viability [287]. Pharmacological inhibition of fatty acid oxidation induces cell cycle arrest at G0/G1 phase [286]. Finally, CPT1 activity also regulates the production of acetyl groups which are used for histone acetylation and thereby control cell growth [288]. Collectively, these studies highlight a complex and diverse array of mechanisms by which CPT1 influences cells cancer cell viability.

Most studies exploring the links between mitochondrial fatty acid oxidation and cancer cell behavior have used etomoxir, which is an irreversible inhibitor of CPT1. Recently, a novel mechanism by which etomoxir inhibits CPT1 activity was reported, whereby etomoxir disrupts the interaction between CPT1A and Rab14, which localizes to the lipid droplet [289]. This study demonstrated the CPT1A-Rab14 interaction likely forms contact sites between mitochondria and lipid droplets, to provide fatty acids for mitochondrial metabolism. While the use of etomoxir is very common, it is not common that the rate of fatty acid oxidation is reported, and the importance of this is exemplified by reports that breast cancer cell proliferation was not altered when fatty acid oxidation was inhibited by 90% by 10 μM etomoxir; it was only at doses ≥ 200 μM of etomoxir that breast cancer cell proliferation was impaired [290]. This study highlighted that etomoxir has an off-target effect at commonly used dosages, including inhibiting complex I of the electron transport chain. Further, the authors also demonstrated that CPT1 regulates other aspects of mitochondrial biology beyond β-oxidation, including supplying the mitochondria with long-chain fatty acyl-CoAs for glycerophospholipid remodeling and protein acylation that are essential for healthy mitochondrial function and cancer cell proliferation [290]. These observations suggest that not all intra-mitochondrial fatty acyl-CoAs enter the β-oxidation pathway but also act as substrates for complex lipid synthesis and acylation reactions within mitochondria.

Downstream of CPT1 is the transfer of fatty acyl-carnitines across the inner mitochondrial membrane by CACT. This is followed by the conversion of fatty acyl-carnitines back to fatty acyl-CoAs by CPT2, which has received very little attention, even though there is only one isoform, unlike CPT1. Protein levels of CPT2 are increased in prostate cancer [291], and pharmacological inhibition or genetic loss-of-function impaired cell growth [277, 291].

Now that fatty acyl-CoAs are in the mitochondrial matrix, they can be substrates for β-oxidation. The oxidation of saturated fatty acyl-CoAs is relatively straightforward, involving involves four consecutive reactions: (i) desaturation of the bond between C2 and C3 by the FAD-dependent acyl-CoA dehydrogenase (ACAD) family; (ii) hydration of the formed 2-enoyl-CoA by enoyl-CoA hydratase; (iii) dehydrogenation of 3-hydroxyacyl-CoA by hydroxyacyl-CoA dehydrogenase; and finally (iv) thiolytic cleavage of 3-oxoacyl-CoA by 3-ketoacyl-CoA thiolase (Fig. 4) [292]. These reactions shorten the fatty acyl-CoA by two carbons between carbons 2 and 3 to produce a shorten acyl-CoA and acetyl-CoA, which the latter is used as a substrate for the TCA cycle. Each cycle also produces one FADH_2_ and one NADH that are reducing equivalents that fuel the electron transport chain to produce ATP.

The presence of one or more double bond introduces complexity into the oxidation of these monounsaturated or polyunsaturated fatty acyl-CoAs (Fig. 4). As an example, oleoyl-CoA contains a double bond between the 9^th^ and 10^th^ carbon and undergoes three cycles of β-oxidation until its double bond is “exposed.” The double bond is removed by Δ^3^, Δ^2^-enoyl-CoA isomerase (encoded by *ECI1*) and the resulting saturated acyl-CoA re-enters the β-oxidation pathway. PUFA catabolism also requires the “removal” of the double bonds as well as the repositioning of specific double bonds. An example is the oxidation of linoleoyl-CoA (linolenic acid; C18:2 (n-9, 12)). These steps involve Δ^3^, Δ^2^-enoyl-CoA isomerase to “remove” the first double between carbons 9 and 10, while the bond between carbon 11 and 12 (which at this point of oxidation is now carbon 4 and 5) is firstly dealt with by 2,4-dienoyl CoA-reductase (encoded by *DECR1*), using NADPH as a co-factor, to form an acyl-CoA with one double bond between carbon 2 and 3 that is then removed by Δ^3^, Δ^2^-enoyl-CoA isomerase, producing a saturated acyl-CoA as a substrate for β-oxidation.

In general, a small number of studies have explored the role of enzymes of mitochondrial β-oxidation, compared to CPT1. The ACAD family of enzymes contains four closely related, chain length-specific acyl-CoA dehydrogenases, including very long-chain, long-chain, medium-chain, and short-chain ACADS; ACADVL, ACADL, ACADM, and ACADS, respectively. ACADL is downregulated in HCC and overexpression results in reduced in vitro cell growth and in vivo tumor size [293]. Conversely, ACADL is upregulated in esophageal squamous cell carcinoma cell lines and tumor tissue and predicts worse outcomes [294]. Enoyl-CoA hydratase catalyzes the second step of mitochondrial β-oxidation and is upregulated and downregulated in a range of cancers (see review [295]). More recently, the reduction in fatty acid, and branched-chain amino acid, oxidation as a consequence of downregulation of enoyl-CoA hydratase leads to lipid accumulation in clear cell renal cell carcinoma, but also results in mTOR activation and cell proliferation [296, 297]. Collectively, these observations highlight a complex role for mitochondrial β-oxidation of long-chain fatty acids, beyond the abundance of CPT1 in tumor fatty acid metabolism.

Alterations in the genes encoding key enzymes that regulate the levels or oxidation status of PUFAs have been reported, and are often closely linked to ferroptosis, as PUFA oxidation is the major cellular stimulus for this iron-dependent form of programmed cell death. Addition of PUFAs, but not other FAs, to cancer cells markedly sensitizes them to induction of ferroptosis [298] due to their high susceptibility to oxidative damage. This can occur enzymatically via the action of lipoxygenases (ALOX1-6), which catalyze deoxygenation of PUFAs to form lipid hydroperoxides, or as discovered in a lentiviral screen of genes that suppress ferroptosis, the catalytic subunit of the phosphorylase kinase complex, PHKG2 [298] which, when inhibited, prevents the formation of lipid hydroperoxides. Interrogation of clinical tissue-derived datasets has revealed that two of the enzymes involved in the auxiliary pathway of PUFA beta-oxidation, ECI2 and the rate-limiting enzyme DECR1, are overexpressed in human prostate cancers [299–301] and associated with poorer overall patient survival [299, 300]. Selective knockdown of these enzymes impacts growth and tumorigenicity of prostate cancer cells, but not non-malignant lines, coincident with an accumulation of cellular PUFAs, resulting in increased lipid peroxidation and induction of ferroptosis [300, 301]. Androgenic regulation of these enzymes [299, 300] further emphasizes their potential importance to prostate tumorigenesis. These effects, however, appear to be cancer type-specific, with DECR1 shown to be decreased in mouse models of breast cancer and in clinical breast tumors compared to normal mammary gland [302, 303], and ectopic expression of DECR1 in HER2/neu-transformed breast cancer cells reducing tumorigenesis—an effect linked to reduced de novo lipogenesis [303]. The future pharmacological modulation of these enzymes, which currently lack small molecule inhibitors or activators, offers the interesting potential to selectively influence PUFA oxidation, compared to broad-spectrum fatty acid oxidation inhibitors of CPT1 for example.

Very-long chain fatty acyl-CoAs (≥ C22) undergo peroxisomal β-oxidation to shorten the fatty acyl-CoAs into smaller units before they are transferred to mitochondria (Fig. 4). Briefly, this process involves the transportation of very long-chain fatty acyl-CoAs into the peroxisome by the peroxisomal ATP-binding cassette (ABC) transporter subfamily D. Very-long chain fatty acyl-CoAs then enter the peroxisomal β-oxidation pathway which consists of 4 steps: (1) oxidation, (2) hydration, (3) dehydrogenation, and (4) thiolysis (see review [304]). The interaction between peroxisomes and mitochondria, including the transfer of shortened fatty acyl-CoAs (~ 8–10 carbons long) and acetyl-CoA, is highly complex [305]. Briefly, fatty acyl-CoAs are converted to acylcarnitines by peroxisomal carnitine octanoyltransferase and transported out of the peroxisome, then into the mitochondria by CACT, where they are then a substrate for mitochondrial CPT2. Peroxisomal acetyl-CoA can either be converted to acetylcarnitine by carnitine acetyltransferase or hydrolyzed by peroxisomal ACOTs and then transferred out of the peroxisomes.

The literature reports varying patterns of peroxisomal gene and protein levels and metabolic flux in cancer cells compared to normal cells (see review [304]). For example, many studies report reduced peroxisomal protein abundance or enzymatic activities in colon, breast, and hepatocellular carcinoma, whereas others have reported *PEX2* mRNA, which encodes peroxisomal biogenesis factor 2 that is required for peroxisome biogenesis, is increased in hepatocellular carcinoma and that knockdown of *PEX2* reduced tumor formation (see review [306]). The complex role of peroxisomes in cancer, including ROS balance and non-β-oxidation pathways, has been recently reviewed [304, 306]. In terms of peroxisomal β-oxidation of very long-chain fatty acyl-CoAs, the expression of the four members of the ABCD transporter family differs between tumor and normal tissue, and between cancer types [307]. For example, ABCD1 is upregulated in breast cancer, unchanged in colorectal and downregulated in melanoma, whereas ABCD2 is downregulated in breast and colorectal cancer [307]. The oxidation step in peroxisomes is catalyzed by acyl-CoA oxidases (ACOX), and to date, there is very little functional understanding of ACOXs in cancer; likewise, the other enzymes of peroxisomal β-oxidation include D-bifunctional protein (DBP, encoded by *HSD17B4*), peroxisomal 3-ketoacyl-CoA thiolase (encoded by *ACAA1*), or sterol-carrier protein X (SCPx). Similarly, enzymes involved in auxiliary pathways including peroxisomal 2,4-dienoyl CoA reductase (DECR2), which is related to mitochondrial DECR1, peroxisomal Δ^3^, Δ^2^-enoyl-CoA isomerases, and downstream export processes catalyzed by peroxisomal carnitine octanoyltransferase (COT), carnitine acetyltransferase (CAT), and ACOTs (see reviews [305, 308]) are currently not well described in cancer. To date, gene expression analysis shows that many of the genes involved in peroxisomal fatty acid metabolism are increased in breast cancer (reviewed in [304]). However, it is not clear whether these changes in gene expression results in altered fatty acid metabolism.

## Lipid profiles as a readout of fatty acid metabolism

We have attempted to discuss lipid synthesis pathways, but it is clear from the recent explosion of the use of mass spectrometry and imaging-based lipidomic platforms to determine the complex lipid and/or fatty acid composition of cancer cells and/or tumors, both at the lipid class level (i.e., PC, PI, TG, etc.) and species level (i.e., TG(16:0/18:1(9Z)/18:0)), that another level of complexity emerges (see review [309]). While variation in platforms and protocols precludes precise comparisons between lipids identified in the increasing number of tumor lipidomics studies reported, it is possible to draw some broader conclusions from the consistent observations that have been made. For example, many tumor types exhibit altered ratios of unsaturated lipids compared to their normal tissue counterparts; most commonly a relative increase in MUFA-containing glycerophospholipids with corresponding decreases in saturated FAs and PUFAs [310]. A greater proportion of MUFAs can, among other functions, protect tumor cells from the toxic effects of excess saturated FAs or PUFAs, thereby enhancing cell survival. Moreover, integration of lipidomic and transcriptomic data revealed that such lipid changes reflect increased SCD activity in more aggressive liver cancers [309], which may represent a targetable and common vulnerability with inhibitors available [176, 178]. Another remarkably common feature of clinical tumors is an enhanced proportion of longer chain fatty acids in glycerophospholipids compared to normal tissues [199, 202, 204], often reflected in altered expression of one or more ELOVL genes that, when targeted, suppress both elongation and tumor cell survival (see above). Considerable heterogeneity is evident when considering individual lipid species that have been identified as classifiers of malignant tissue (reviewed in [218, 309]), although the increasing adoption of mass spectrometry imaging to link lipid content to histological features of tissues is likely to yield more consistent findings than bulk tumor analysis where altered cellularity may complicate results. Frequently, tumors feature altered levels of lysophospholipids, TGs, PCs, and PIs, which are not only potential biomarkers of malignant tumor areas in surgical applications, but clearly reflect an underlying altered biology that may indicate novel therapeutic approaches that are common to certain cancer types or subsets of patients.

Alongside the advances in our understanding of what defines the tumor lipidome, a small number of studies have identified specificity in the metabolism of fatty acyl species (i.e., saturate, MUFA, PUFA) that point toward changes in substrate specificity rather than just bulk fatty acid metabolism. Firstly, PUFAs are sequestered into lipids to reduce PUFA-induced lipotoxicity and susceptibility to ROS attack, thereby promoting triple-negative breast cancer cell survival [42]. Significantly, preventing the mobilization of PUFAs from the lipid droplet, by inhibition of ATGL, reduced PUFA-induced oxidative stress and cell death. On the other hand, MUFAs are mobilized from TGs stored in lipid droplets, via the actions of HSL, in response to hypoxia and nutrient stress to maintain appropriate fatty acid balance between saturated and unsaturated fatty acids in ccRCC cells [122]. The reduced availability of oxygen, a co-factor for SCD production of MUFAs, results in the production of lipotoxic ceramides that contain saturated fatty acyl species leading to reduced cancer cell survival. These recent observations, in combination with lipidomic studies described above, point to important roles for specific fatty acid species, beyond eicosanoid production, where changes in the MUFA/PUFA and saturated/MUFA ratios can have profound effects on cell function, including activation of ferroptosis, ceramide synthesis, ER stress, and apoptosis as well as increased sensitivity to chemotherapeutic agents. Readers are pointed toward a recent comprehensive review of the specific effects of saturated, MUFA, and PUFA in cancer cell biology for additional insights [311].

The changes in MUFA/PUFA and saturated/MUFA ratios are reflected in the fatty acyl side chains of membrane lipids that influence cellular function. Cellular membranes are highly organized, and so introduce another next level of complexity that must be acknowledged. Specifically, the plasma membrane has asymmetrical lipid distribution, whereas the endoplasmic reticulum membrane and others are symmetrical. The symmetry of membranes is controlled by active transporters, including flippases and floppases, and passive scramblases (Fig. 1) [312]. Then add to this the very recent report that the plasma membrane exhibits dramatic glycerophospholipid unsaturation asymmetry with the cytoplasmic leaflet being approximately twofold more unsaturated than the exoplasmic leaflet [313]. Adding further complexity is the role of subdomain features of plasma membranes such as lipid raft domains, which is a topic that was recently reviewed [314]. Readers are also pointed toward a detailed review of the broader topics of lipid topogenesis for further insight into this highly complex area of cell biology [315]. Collectively, the synthetic pathways of fatty acid metabolism have a direct influence on a range of features of cell membrane chemistry, such as saturation and chain length, that then influences the biophysical properties of membranes including fluidity, curvature, and subdomain architecture [26]. In turn, these factors influence membrane-associated cellular processes, such as vesical trafficking, signal transduction, and molecular transport that can influence cell proliferation and viability [316].

## Aspects of tumor fatty acid metabolism that warrant closer attention

We have described the breadth of fatty acid metabolism in a reductionist manner but believe that there are some complex issues that, if resolved, will assist the interpretation of future studies. The first aspect is compartmentalization. We have highlighted that there is no consistent pattern of accumulation or depletion of intermediates of glycerolipid and glycerophospholipid synthesis (i.e., LPA, PA, DG) in cancer cells compared to non-cancer controls (see “Simple and complex lipid synthesis” section). This may be, in part, attributed to these intermediates existing in multiple subcellular pools that are distinctly regulated. A key example is the total cellular DG, which is an integrated readout of DG in the plasma membrane, endoplasmic reticulum, and lipid droplets pools, if not all organelle membranes. Each of these pools is regulated by distinct processes, and this subcellular localization information is lost when measuring lipid levels in cell/tissue homogenates, either by biochemistry or mass-spectrometry based lipidomics.

Secondly, many enzymes described in this review exhibit affinity for multiple and diverse substrates. For example, DGATs not only have an affinity for DGs, but it was recently identified that DGAT2 has ceramide acyltransferase activity to synthesis 1-O-acylceramide in HCT116 colorectal carcinoma cells [117]. ABHD5, which regulates ATGL activity via protein-protein interaction, possesses LPAAT activity, converting lysophosphatidate into phosphatidate [137]. Likewise, several proteins involved in glycerolipid synthesis influence cell biology through non-lipid catalyzing mechanisms. LPIN1, as an example, has phosphatidate phosphatase activity to catalyze the conversion of phosphatidate to DG, but also directly interacts with insulin receptor substrate 1 to influence IGF-1/IR signaling [317] and regulates the expression of key fatty acid oxidation gene [222]. Likewise, G0S2 functions as a tumor suppressor in part by opposing MYC activity [318], alongside its role as a regulator of ATGL activity. Since many loss-of-function experiments of enzymes of the glycerolipid and glycerophospholipid synthesis pathways fail to report the lipid profile of cells, it is difficult to ascribe the changes in cell growth to alterations in lipid levels. As such, linking changes in enzyme expression to variations in specific lipid levels, especially when enzymes have multiple substrates that each may influence proliferation and viability, remains a major issue in the field.

Nearly all of the enzymes we have discussed exist as isozymes, which introduce potential problems of redundancy, an additional layer of specificity through their subcellular localization and/or substrate specificity as determined by fatty acyl species (saturated, MUFA, PUFA, etc.). For example, the GPAT family comprises of four isoforms (GPAT1-4) that reside in specific subcellular locations, such as GPAT1 and 2 on the outer mitochondrial membrane, whereas GPAT3 and 4 localize to the ER [319]. The ELOVL family consists of seven members (ELOVL1-7) with each isozyme exhibiting distinct but overlapping substrate specificity for specific chain-lengths and/or degree of saturation [320]. Additionally, the post-translational and allosteric regulation of many enzymes that participate in fatty acid metabolism remains to be determined. As such, there is a long way to go before we fully understand the various levels of regulation of cellular fatty acid metabolism and how this is altered in cancer.

Another aspect is the existence of plasticity within cellular fatty acid metabolism, due to the inter-connectivity of the pathways. A recent example is that the rate of de novo fatty acid synthesis is upregulated in response to SR-B2/CD36 inhibition to reduce the uptake of extracellular fatty acids [52]. Likewise, ACSS2, which produces acetyl-CoA from acetate, is upregulated in response to reduced acetyl-CoA production from citrate due to ACLY silencing [321]. As such, inhibition of key enzymes of fatty acid metabolism is likely to result in adaptation because of multiple substrate sources and the interconnection of downstream pathways.

Finally, the selection of experimental conditions and model systems used has likely hampered progress in our understanding of tumor fatty acid metabolism. To date, much of the field has explored cancer cell lipid metabolism in monolayer cell culture, and the emergence of technologies including 3-dimensional cell culture, microfluid cell culture, and organs-on-a-chip as well as tumor explant cultures [322], all provide the opportunity to define metabolic pathways in, what are perceived to be, more physiologically relevant models. Alongside model selection, experimental conditions and media selection are other areas that are gaining awareness, including physiologic media that more closely resembles human or rodent plasma compared to traditional cell culture media [323]. While the current focus of these physiological media rightly focused on amino acid and glucose levels [324], there remains a significant challenge to have these media reflect the physiological lipid environment. This issue already exists with existing cell culture approaches, which was comprehensively reported recently by Prof Else, where he concluded that “Fetal bovine serum… at 10% of media provides 2–3% of the fatty acid and cholesterol, 1% of the PUFA and 0.3% of the essential fatty acid linoleic acid (18:2n-6) available to cells in the body” [325]. As we have highlighted in this review, lipid metabolism is critical for cancer cell biology and so significant consideration must be made when attempting to design “physiological media” that truly reflects the levels of all nutrients, especially the diversity of extracellular lipid sources (e.g. lipoproteins, albumin-bound or FABP4-bound free fatty acids, etc.).

## Conclusions

It has been long appreciated that the various endpoints of fatty acid metabolism, including energy production (β-oxidation), synthesis of signaling lipids, altered protein function via acylation, and membrane lipid synthesis and modification to alter fluidity and permeability, can profoundly influence tumor progression, including treatment resistance. It is for these reasons that there are many inhibitors of fatty acid metabolism that are currently under development or undergo clinical trial testing (see recent reviews for summaries [218, 326, 327]). In the last 5 years, there have been significant advances in our understanding of the role that fatty acid metabolism plays in many aspects of tumor biology. These include a more precise definition of processes that act as input sources to the intracellular fatty acid pool and the many outputs of the fatty acyl-CoA pool, including those endpoints list above. Further, the field has gained more nuanced insights into tumor fatty acid metabolism, identifying key differences between saturated fatty acid, MUFA and PUFA metabolism that participate in the maintenance of cellular homeostasis including in the management of redox stress, thereby preventing anoikis and ferroptosis. Likewise, fatty acid chain length is emerging as an underappreciated aspect of fatty acid metabolism that may be an exploitable vulnerability. That said, the bulk availability of fatty acids is likely to remain an influencing factor and thereby targetable strategy for cancer control; however, it is highly likely that the diverse sources of fatty acid available to cancer cells likely provide redundancy and thereby will challenge the effectiveness of suppressing a single pathway. A significant challenge that the field faces remains the ability to identify the mechanism(s) by which changes gene/protein levels that (should) alter lipid levels influence cell biology. It is one that the development and deployment of more sensitive technologies may help overcome.

## Data Availability

Not applicable.

## Abbreviations

ABC: ATP-binding cassette
ACAD: Acyl-CoA dehydrogenase
ACAT: Acyl-CoA:cholesterol acyltransferases
ACBD: Acyl-CoA-binding domain-containing proteins
ACC: Acetyl-CoA carboxylase
ACLY: ATP-citrate lyase
ACOT: Acyl-CoA thioesterases
ACOX: Acyl-CoA oxidase
ACS: Acyl-CoA synthetase
ACSS2: Acyl-coenzyme A synthetase short-chain family member 2
AGPAT: Acylglycerol-3-phosphate acyltransferase
ATGL: Adipose triacylglycerol lipase
C: Carbon
CACT: Carnitine-acylcarnitine translocase
CAT: Carnitine acetyltransferase
CD36: Cluster of differentiation 36
CDCP1: CUB-domain containing protein 1
CES: Carboxylesterase
COT: Carnitine octanoyltransferase
CPT: Carnitine palmitoyltransferase
DBP: D-bifunctional protein
DECR: 2,4-Dienoyl CoA reductase
DG: Diacylglycerol
DGK: Diacylglycerol kinases
ELOVL: Elongation of very long-chain fatty acid enzyme
FABP: Fatty acid-binding protein
FAS: Fatty acid synthase
FATP: Fatty acid transport protein
G0S2: G0/G1 switch gene 2
GPAT: Glycerol-3-phosphate acyltransferase
HIG2: Hypoxia-inducible gene 2
HILPDA: Hypoxia-inducible lipid droplet associated protein
HRASLS: H-RAS-like suppressor
HSL: Hormone-sensitive lipase
LDL: Low-density lipoprotein
LDLr: Low-density lipoprotein receptor
LPAAT: Lysophosphatidate acyl transferase
LPCAT1: Lysophosphatidylcholine acyltransferase 1
LPL: Lipoprotein lipase
LPLAT: Lysophospholipid acyltransferase
LYPLA: Lysophospholipase A
LPR1: Low-density lipoprotein receptor-related protein 1
LSR: Lipolysis-stimulated receptor
MBOAT: Membrane-bound O-acyltransferase
MG: Monoacylglycerol
MOGAT: Monoacylglycerol acyltransferase
mTORC2: mTOR Complex 2
MUFA: Monounsaturated fatty acid
PC: Phosphatidylcholine
PDK1: Phosphoinositide-dependent kinase-1
PE: Phosphatidylethanolamine
PG: Phosphatidylglycerol
PI: Phosphatidylinositol
PLA: Phospholipase A
PLB: Phospholipase B
PNPLA: Phospholipase domain-containing protein
PTEN: Phosphatase and tensin homolog
PUFA: Polyunsaturated fatty acid
ROS: Reactive oxygen species
SCD: Stearoyl-CoA desaturase
SOAT: Sterol O-acyltransferase
SR-B2: Scavenger receptor B2
TG: Triacylglycerol
VLDL: Very low-density lipoprotein
VLDLr: Very low-density lipoprotein receptor
